# TGFβ-Induced Deptor Suppression Recruits mTORC1 and Not mTORC2 to Enhance Collagen I (α2) Gene Expression

**DOI:** 10.1371/journal.pone.0109608

**Published:** 2014-10-15

**Authors:** Falguni Das, Amit Bera, Nandini Ghosh-Choudhury, Hanna E. Abboud, Balakuntalam S. Kasinath, Goutam Ghosh Choudhury

**Affiliations:** 1 Departments of Medicine, University of Texas Health Science Center at San Antonio, San Antonio, Texas, United States of America; 2 Department of Pathology, University of Texas Health Science Center at San Antonio, San Antonio, Texas, United States of America; 3 Geriatric Research, Education and Clinical Center, South Texas Veterans Health Care System, San Antonio, Texas, United States of America; 4 VA Research, South Texas Veterans Health Care System, San Antonio, Texas, United States of America; Children's Hospital Boston & Harvard Medical School, United States of America

## Abstract

Enhanced TGFβ activity contributes to the accumulation of matrix proteins including collagen I (α2) by proximal tubular epithelial cells in progressive kidney disease. Although TGFβ rapidly activates its canonical Smad signaling pathway, it also recruits noncanonical pathway involving mTOR kinase to regulate renal matrix expansion. The mechanism by which chronic TGFβ treatment maintains increased mTOR activity to induce the matrix protein collagen I (α2) expression is not known. Deptor is an mTOR interacting protein that suppresses mTOR activity in both mTORC1 and mTORC2. In proximal tubular epithelial cells, TGFβ reduced deptor levels in a time-dependent manner with concomitant increase in both mTORC1 and mTORC2 activities. Expression of deptor abrogated activity of mTORC1 and mTORC2, resulting in inhibition of collagen I (α2) mRNA and protein expression via transcriptional mechanism. In contrast, neutralization of endogenous deptor by shRNAs increased activity of both mTOR complexes and expression of collagen I (α2) similar to TGFβ treatment. Importantly, downregulation of deptor by TGFβ increased the expression of Hif1α by increasing translation of its mRNA. TGFβ-induced deptor downregulation promotes Hif1α binding to its cognate hypoxia responsive element in the collagen I (α2) gene to control its protein expression via direct transcriptional mechanism. Interestingly, knockdown of raptor to specifically block mTORC1 activity significantly inhibited expression of collagen I (α2) and Hif1α while inhibition of rictor to prevent selectively mTORC2 activation did not have any effect. Critically, our data provide evidence for the requirement of TGFβ-activated mTORC1 only by deptor downregulation, which dominates upon the bystander mTORC2 activity for enhanced expression of collagen I (α2). Our results also suggest the presence of a safeguard mechanism involving deptor-mediated suppression of mTORC1 activity against developing TGFβ-induced renal fibrosis.

## Introduction

Renal tubulointerstitial fibrosis represents the best predictor of clinical outcome of end-stage renal disease [1]. The initiation of phase of fibrosis involves infiltration of inflammatory cells that secrete profibrogenic growth factors and cytokines. One such factor, TGFβ, acts on various renal cells including the proximal tubular epithelial cells to increase expression of matrix proteins, which significantly contribute to the fibrotic process. TGFβ through binding to the type II receptor engages the FKBP12-bound type I receptor to induce heterotetramerization, increase in phosphorylation of type I receptor and release of FKBP12 [2], [3]. Activated type I receptor then phosphorylates the receptor-specific Smads (Smad 3 and 2) at the C-terminus, which is then released from the type I receptor and SARA, a Smad-recruiting protein to the plasma membrane [4]. Subsequently, the receptor-specific Smads heterodimerize with co-Smad, Smad 4, and translocate to the nucleus to bind to transcriptional coactivators or corepressors to regulate gene expression [5], [6], [7]. Although Smad 2 and 3 act downstream of TGFβ receptor function, a recent study indicated a protective function of Smad 2 in renal fibrosis and matrix protein expression in proximal tubular epithelial cells [8].

Apart from canonical Smad signaling, TGFβ has been shown to induce many kinase cascades that are known to be activated by receptor tyrosine kinases, such as Erk1/2, JNK1/2, p38 MAPK and c-Src tyrosine kinase [7], [9], [10]. Furthermore, TGFβ activates PI 3 kinase and Akt to regulate renal pathology including renal cell hypertrophy and fibrosis [11], [12], [13], [14]. Recently, we and others have shown activation of mTOR kinase in response to TGFβ [15], [16], [17], [18]. In mammals, mTOR exists in two distinct complexes mTORC1 and mTORC2, which differ in their compositions. Raptor is only present in mTORC1 while both rictor and Sin1 define mTORC2 [19], [20], [21]. The regulation of mTORC1 and mTORC2 catalytic activity is complex. For example, raptor, the exclusive component of mTORC1, is phosphorylated by mTORC1 to increase its activity [22]. However, mTORC1 impairs activation of mTORC2 by phosphorylation of IRS-1 and Grb-2, which are involved in PI 3 kinase signaling [21], [23], [24]. On the other hand, mTORC2-mediated phosphorylation of Sin1 increases its stability by inhibiting its lysosomal degradation to maintain the mTORC2 activity [25]. In contrast to these results, a recent report established the mTORC1-activated S6 kinase-dependent inhibitory phosphorylation of Sin1 at Thr-86 and Thr-398, which are present in the N-and C-terminal domains necessary for interaction with rictor and mTOR, respectively [26]. The sensitivity of mTORC1 and mTORC2 to the macrolide rapamycin and substrate specificities differ significantly [20]. mTORC1 essentially regulates the anabolic program of the cells by controlling protein synthesis, mitochondrial biogenesis, lipogenesis and nucleotide biosynthesis [19], [27]. On the other hand, mTORC2 controls cytoskeletal organization, cell survival, gluconeogenesis and lipogenesis by activating AGC kinases and inactivating class IIa histone deacetylases, respectively [20], [21], [28], [29]. However, using rapamycin we and others have recently shown involvement of mTOR to contribute to renal cell pathology found in diabetic kidney disease including kidney hypertrophy and matrix protein expression [30], [31], [32], [33], [34]. More recently, we identified a role for both mTORC1 and mTORC2 in TGFβ induction of renal cell matrix protein synthesis [5], [15], [16], [35]. The precise mechanism by which mTOR is activated to increase matrix protein expression is not known. Here we show that deptor, a recently identified negative regulatory component of both mTORC1 and mTORC2, contributes to TGFβ-induced matrix protein collagen I (α2) expression in human proximal tubular epithelial cells. We demonstrate that downregulation of deptor by TGFβ is necessary for collagen I (α2) expression. Furthermore, we show that deptor inhibits TGFβ-induced Hif1α, which binds to the collagen I (α2) promoter to induce its transcription. We demonstrate that deptor regulates Hif1α mRNA translation to increase its protein levels. Finally, we show that in type 2 diabetic mice, increased expression of TGFβ is associated with decreased deptor expression and enhanced Hif1α, and collagen I (α2) levels. Thus our results demonstrate a significant role of deptor in regulating collagen I (α2) expression in TGFβ-mediated fibrotic response.

## Materials and Methods

### Materials

TGFβ1 was purchased from R & D, Minneapolis, MN. NP-40, Na_3_VO_4_, phenylmethylsulfonylfluoride, protease inhibitor cocktail, anti-FLAG (M5) and β-actin antibody were obtained from Sigma, St Louis, MO. Antibodies against phospho-S6 kinase (Thr-389), S6 kinase, phospho-4EBP-1 (Thr-37/46), 4EBP-1, phospho-Akt (Ser-473), phospho-Akt (Thr-308), phospho-tuberin (Thr-1462), phospho-PRAS40 (Thr-246), Akt, raptor, rictor and PRAS40 were purchased from Cell Signaling, Boston, MA. Antibodies against Hif1α, deptor, tuberin and collagen I (α2) and siRNA against Hif1α and scramble RNA were obtained from Santa Cruz Biotechnology, Delaware, CA. Detailed description of the antibodies is presented in Table S1. Tissue culture materials, cDNA synthesis kit and TRI reagent for RNA isolation, were obtained from Invitrogen, Carlsbad, CA. FuGENE HD transfection reagent and luciferase assay kit were purchased from Promega Inc. Madison, WI. RT^2^ real-time SYBR green/ROX PCR mix and GAPDH primers were obtained from Qiagen. Luciferase assay kit was obtained from Promega. Plasmids containing FLAG-tagged deptor and deptor shRNAs (deptor sh1 and deptor sh2) were constructed in the laboratory of Dr. David Sabatini, Whitehead Institute for Biomedical Research, Boston, MA and were obtained from Addgene. Vectors containing scramble RNA, raptor shRNA and rictor shRNA were used previously [16]. Hif1α 5′ terminal oligopyrimidine tract (TOP)-Lux reporter plasmid was constructed in the laboratory of Dr. Charles Sawyers, University of California at Los Angeles and was obtained from John Blenis (Harvard Medical School) [36].

### Cell Culture and Treatment

The HK2 human kidney proximal tubular epithelial cells were grown in DMEM/F12 medium with 10% fetal bovine serum as described previously [37]. The mouse proximal tubular epithelial cells were grown in DMEM with low glucose in the presence of 7% fetal bovine serum [38]. The cells were starved in serum free medium for 24 hours and incubated with 2 ng/ml TGFβ for indicated periods of time.

### Cell Lysis and Immunoblotting

Incubated cells were washed twice with PBS and harvested in RIPA buffer (20 mM Tris-HCl, pH 7.5, 5 mM EDTA, 150 mM NaCl, 1% NP-40, 1 mM Na_3_VO_4_, 1 mM PMSF and 0.1% protease cocktail). The cell monolayer was incubated at 4°C for 30 minutes before it was scraped and centrifuged at 4°C for 20 minutes. The supernatant was collected, protein estimated and equal amounts of protein were separated by SDS polyacrylamide gel electrophoresis. The separated proteins were transferred to PVDF membrane and immunoblotting was carried out using indicated primary antibodies. The proteins were developed with HRP-conjugated secondary antibody using ECL reagent as described [37], [39].

### Real Time Quantitative RT-PCR

Total RNAs were prepared using TRIzol reagent as described previously [37], [38], [40]. First strand cDNAs were made with 1 mg RNA using oligo-dT and M-MuLV reverse transcriptase. The cDNA was amplified in a 96-well plate using collagen I (α2) primers (Forward: 5′- GGTCTGGATGGATTGAAGGGACAGC -3′ and Reverse: 5′-GGCTCCTGTTTGACCTGGAGTTCC -3′) in a 7500 real time PCR machine (Applied Biosystem). The PCR conditions were 94°C for 10 minutes followed by 45 cycles at 94°C for 30 seconds, 58°C for 30 seconds and 72°C for 30 seconds. The level of mRNA was normalized by GAPDH in the same sample. Data were analyzed by comparative C_t_ method as described [38].

### Transfection and Luciferase Assay

Cells were transfected with indicated plasmids or siRNA using FuGENE HD reagent according to vendor's protocol. For reporter assays, the reporter plasmids were transfected along with indicated expression plasmids and siRNA. The transfected cells were incubated with TGFβ as described in the legends to the Figures. The cell lysates were used to assay luciferase activity using a kit [5], [39], [41]. The data are presented as mean of luciferase activity per microgram protein as arbitrary units ± SE of indicated measurements as described in the figure legends [42].

### Chromatin Immunoprecipitation Assay

Cell monolayer was used to prepare sheared chromatin essentially as described previously [43], [44]. Sheared chromatin was incubated with protein G-plus Agarose. The cleared chromatin was used as the input control. Sheared chromatin was then incubated with nonimmune IgG or Hif1α antibody to immunoprecipitate the Hif1α-bound DNA fragment along with protein G-plus Agarose. The eluted DNA from the immunoprecipitates was amplified with collagen I (α2) primers (Forward: 5′-CGAGTCAGAGTTTCCCCTTGAAAGC -3′ and Reverse: 5′-CGCAGAGGAGGGAGCGAATG -3′) spanning the hypoxia responsive element (HRE). The product was analyzed by agarose gel electrophoresis. Also the PCR reaction was carried out in a real time PCR machine as described above. The PCR condition was: 94°C for 10 minutes followed by 40 cycles of 94°C for 30 seconds, 58°C for 30 seconds and 72°C for 30 seconds, respectively.

## Results

### TGFβ downregulates deptor to increase expression of collagen I (α2)

**Figure 1:**
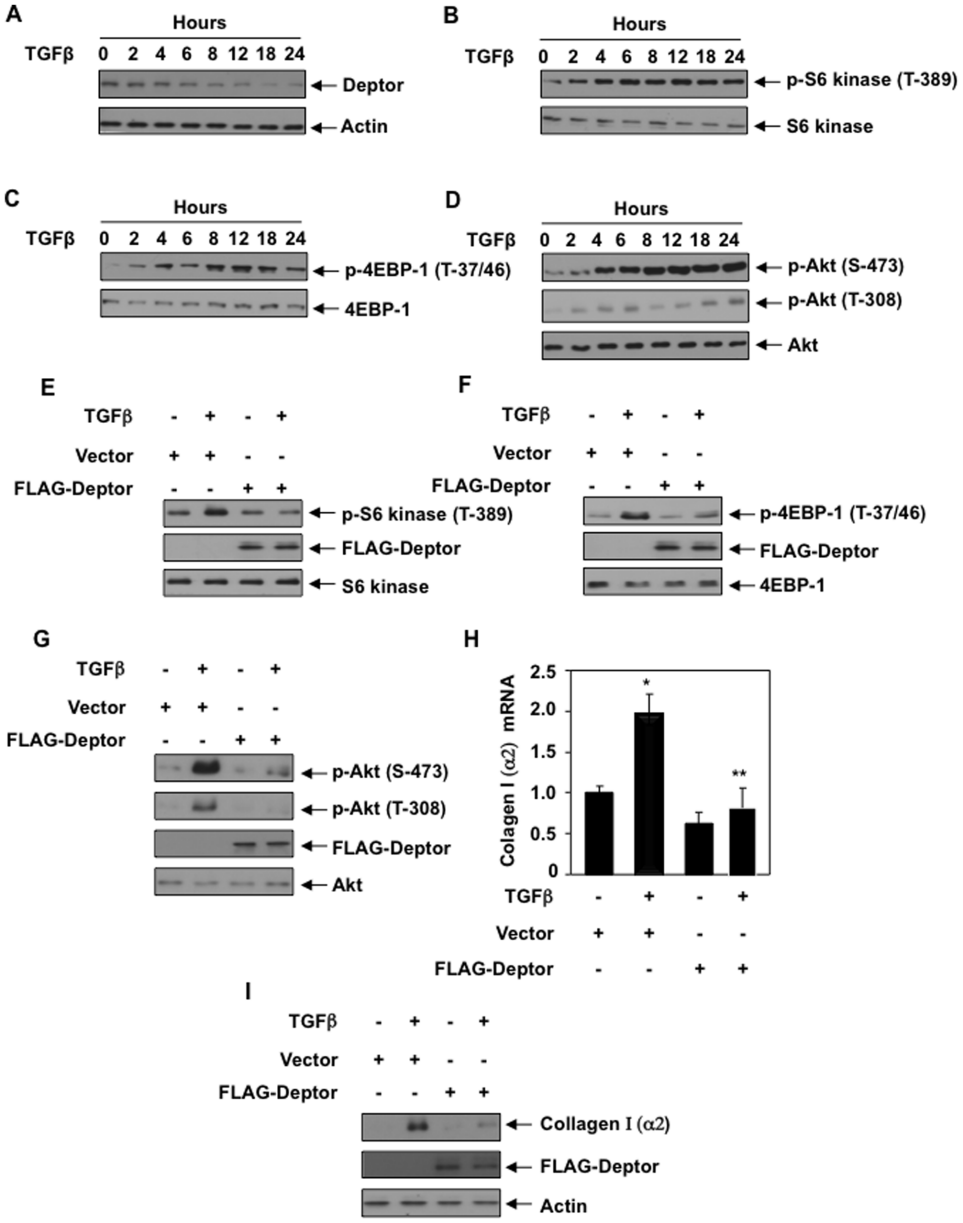
TGFβ-induced suppression of deptor regulates collagen expression in proximal tubular epithelial cells. (A–D) TGFβ decreases deptor resulting in increased mTORC1 and mTORC2 activity. Human proximal tubular epithelial cells were incubated with 2 ng/ml TGFβ for indicated period of time. The cell lysates were immunoblotted with deptor, actin (panel A), phospho-S6 kinase (Thr-389), S6 kinase (panel B), phospho-4EBP-1 (Thr-37/46), 4EBP-1 (panel C) and phospho-Akt (Ser-473), phospho-Akt (Thr-308) and Akt (panel D) antibodies as indicated. (E–G and I) Expression of deptor inhibits mTORC1 and mTORC2 activities to block collagen expression. Human proximal tubular epithelial cells were transfected with FLAG-tagged Deptor expression plasmid or vector. Transfected cells were incubated with 2 ng/ml TGFβ for 24 hours. The cell lysates were immunoblotted with phospho-S6 kinase, S6 kinase (panel E), phospho-4EBP-1, 4EBP-1 (panel F), phospho-Akt, Akt (panel G), collagen I (α2), actin (panel I) as indicated. The same lysates were used to immunoblot with FLAG antibody to demonstrate deptor expression. Quantifications of panels A–G are shown in Fig. S2A–S2G. (H) Expression of deptor inhibits mTORC1 and mTORC2 activities to block collagen mRNA expression. Human proximal tubular epithelial cells were transfected with FLAG-tagged Deptor expression plasmid or vector. Transfected cells were incubated with 2 ng/ml TGFβ for 24 hours. Total RNAs were prepared and used for real time RT-PCR to detect collagen mRNA as described in the Materials and Methods. Mean ± SE of triplicate measurements is shown. *p<0.01 vs control; **p<0.01 vs TGFβ-treated. Expression of Deptor in parallel samples is shown in Fig. S3A. Quantification of panel I is shown in Fig. S3B.

TGFβ increases expression of various matrix proteins including collagen I (α2) in renal proximal tubular epithelial cells. We have shown previously that TGFβ rapidly activates both mTORC1 and mTORC2 [13], [16]. TGFβ–stimulated increase in PI 3 kinase activity precedes the activation of both these complexes [13], [16], [45]. However, prolonged incubation of cells with TGFβ is required for collagen I (α2) expression and rapamycin, a potent inhibitor of mTOR kinase activity, inhibited collagen I (α2) expression (Fig. S1) [17], [46]. Therefore, we investigated the mechanism of prolonged activation of mTOR necessary for collagen I (α2) expression. Recently, a negative regulator of mTOR activity, deptor, has been identified [47]. Deptor is a common component of both mTORC1 and mTORC2. Since deptor maintains the basal activity of both these complexes, we investigated its expression in human proximal tubular epithelial cells, which accumulates collagen in response to TGFβ. Incubation of proximal tubular epithelial cells with TGFβ significantly inhibited the levels of deptor in a time-dependent manner till 24 hours (Fig. 1A and Fig. S2A). As deptor is an inhibitor of mTOR, we examined activation of mTORC1 employing phosphorylation of S6 kinase (Thr-389) and 4EBP-1 (Thr-37/46) as indicators. As shown in Figs. 1B and 1C, TGFβ increased the phosphorylation of S6 kinase and 4EBP-1 at same kinetics (Figs. S2B and S2C) as deptor downregulation. Similarly, TGFβ increased mTORC2 activity as determined by the phosphorylation of its substrate Akt at Ser-473 (Fig. 1D and Fig. S2D) [48]. Note that phosphorylation of Akt at Thr-308 was also increased by TGFb (Fig. 1D). Together, these results suggest that TGFβ-induced deptor downregulation activates both mTORC1 and mTORC2 in a prolonged manner.

**Figure 2:**
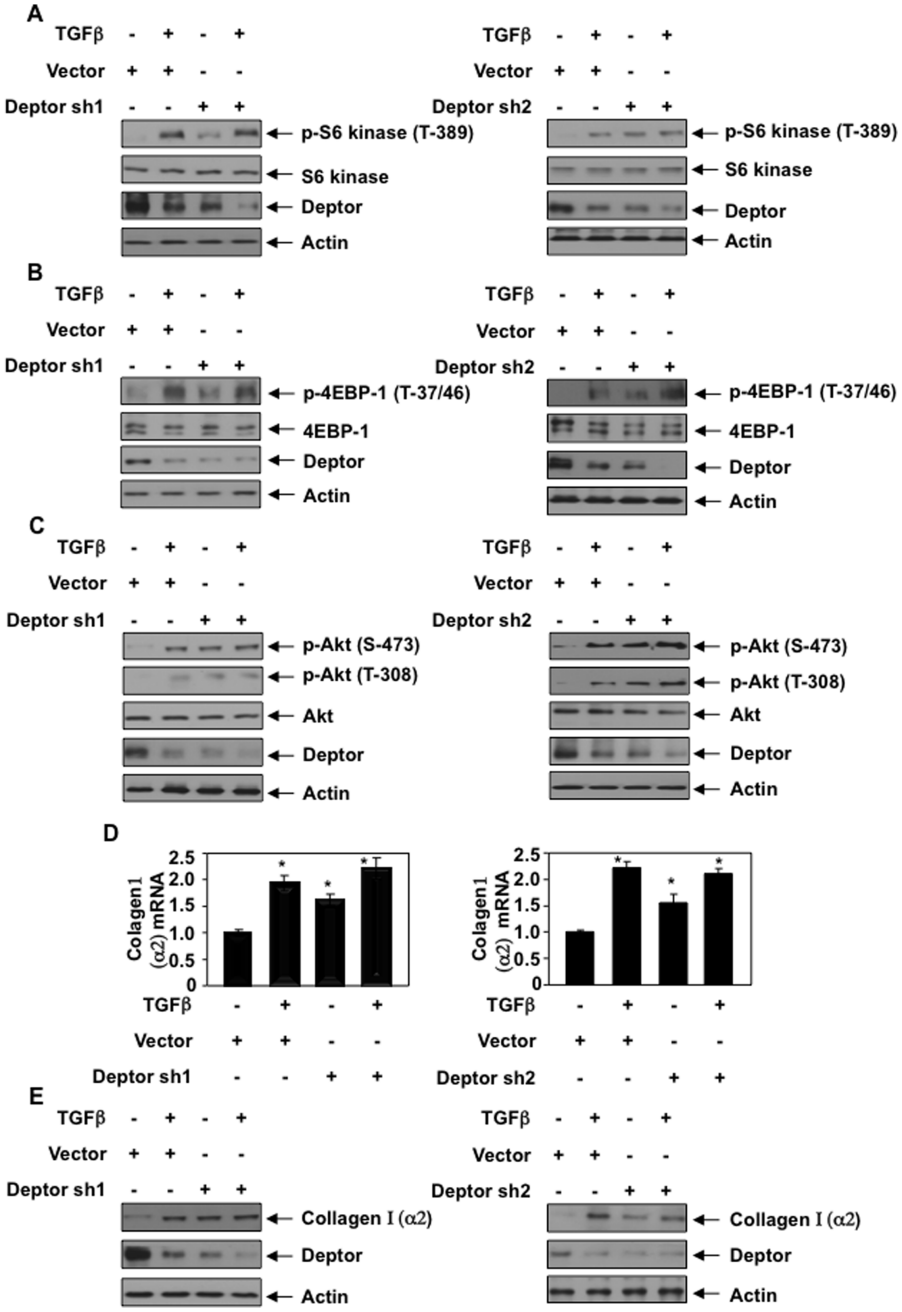
shRNA-mediated repression of deptor increases mTORC1 and mTORC2 activity, resulting in collagen I (α2) expression similar to TGFβ. (A–C and E) Human proximal tubular epithelial cells were transfected with two independent shRNAs against deptor (Deptor sh1 and Deptor sh2). The transfected cells were incubated with 2 ng/ml TGFβ for 24 hours. The cell lysates were immunoblotted with indicated antibodies. Quantifications of panels A–C are shown in Figs. S4A–S4C. (D) Human proximal tubular epithelial cells were transfected with two independent shRNAs against deptor (Deptor sh1 and Deptor sh2). The transfected cells were incubated with 2 ng/ml TGFβ for 24 hours. Total RNA was prepared and used for real time RT-PCR to detect collagen I (α2) mRNA as described in the Materials and Methods. Mean ± SE of triplicate measurements is shown. *p<0.01 vs control. Expression of Deptor in parallel samples is shown in Fig. S5A. Quantification of Fig. 2E is shown in Fig. S5B.

To determine the role of deptor in collagen I (α2) expression, we used FLAG-tagged deptor expression vector in proximal tubular epithelial cells. Expression of deptor significantly inhibited both mTORC1 and mTORC2 activity induced by 24 hours incubation with TGFβ (Figs. 1E–1G and Figs. S2E–S2G). As expected, TGFβ increased the expression of collagen I (α2) mRNA. Expression of deptor significantly inhibited TGFβ-induced collagen I (α2) mRNA expression (Fig. 1H and Fig. S3A). Similarly, deptor attenuated collagen I (α2) protein expression in response to TGFb (Fig. 1I and Fig. S3B). To confirm the role of deptor, we used two independent shRNA vectors against deptor. Expression of both these shRNAs alone in proximal tubular epithelial cells increased phosphorylation of S6 kinase, 4EBP-1 (indicator of mTORC1) and Akt (indicator of mTORC2) similar to TGFβ treatment (Figs. 2A–2C and Fig. S4A–S4C). Importantly, both shRNAs against deptor significantly increased collagen I (α2) mRNA expression similar to that with TGFβ alone (Fig. 2D and Fig. S5A). Similarly, deptor shRNAs alone were sufficient to significantly increase collagen I (α2) protein level (Fig. 2E and Fig. S5B). Deptor shRNAs did not have any significant additive effect when used along with TGFβ treatment (Figs. 2D and 2E), suggesting that TGFβ effects on the tested parameters were mediated via reduction in deptor. To confirm the role of deptor in collagen I (α2) expression and to examine the specificity of deptor shRNA, we used mouse proximal tubular epithelial cells. shRNA against mouse deptor was transfected into these cells followed by incubation with TGFβ. We also performed rescue experiment in these mouse cells by transfecting FLAG-tagged human deptor, which is not recognized by the mouse deptor shRNA. The results show that mouse shDeptor significantly increased collagen I (α2) expression similar to TGFβ treatment (Fig. S5C). Importantly, expression of mouse shDeptor-resistant human deptor inhibited mouse shDeptor-induced increase in collagen I (α2) expression both in the absence and presence of TGFβ (Fig. S5C).

### Deptor regulates collagen I (a2) transcription

**Figure 3:**
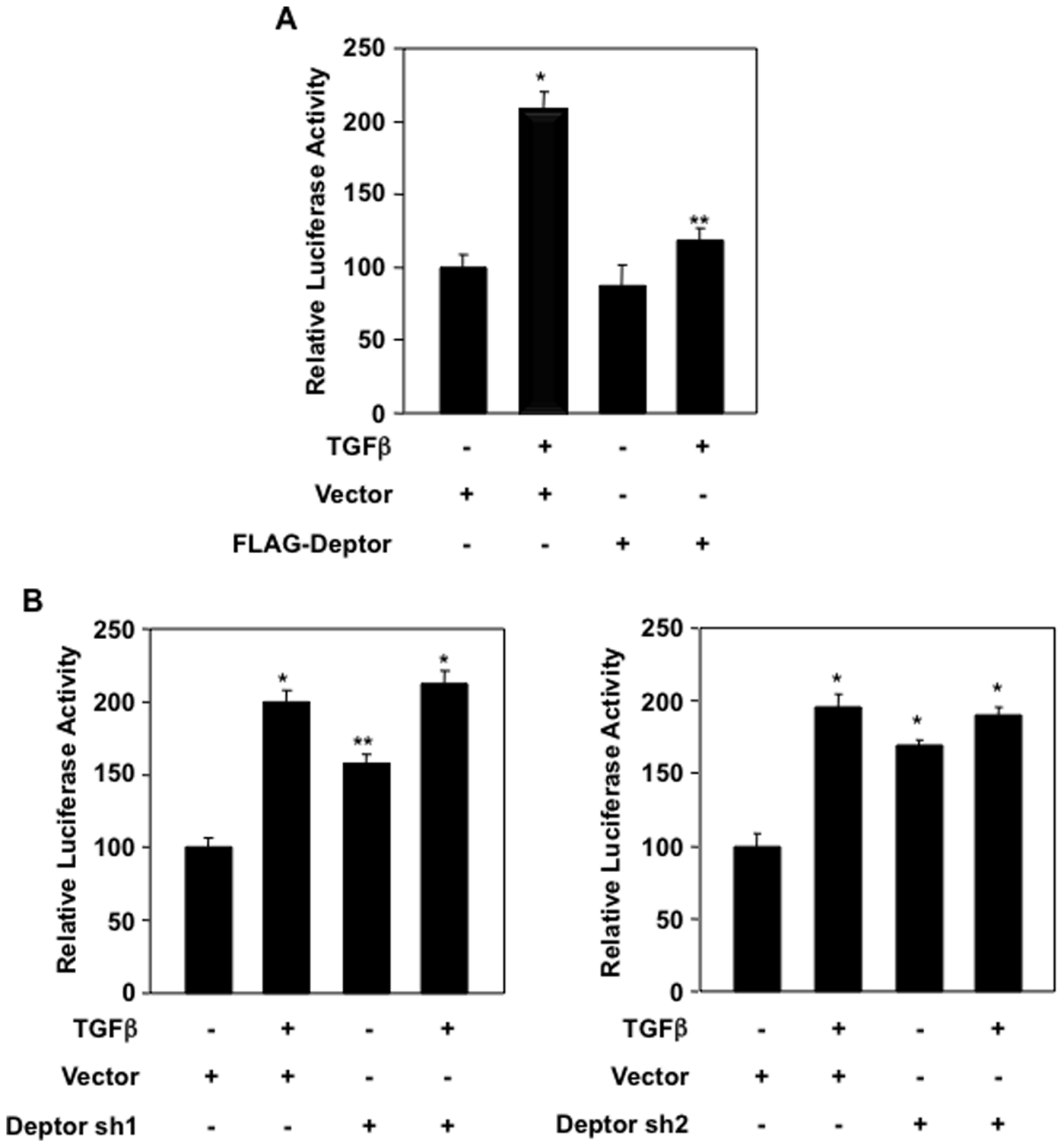
TGFβ-induced deptor downregulation regulates collagen I (α2) transcription. Collagen I (α2 promoter-driven luciferase reporter plasmid was co-transfected with FLAG-deptor (panel A) or shRNAs against deptor (Deptor sh1 and Deptor sh2) (panel B). The transfected cells were incubated with TGFβ for 24 hours. The cell lysates were assayed for luciferase activity as described in the Materials and Methods [5], [39]. Mean ± SE of 3 measurements is shown. For panel A, *p<0.01 vs control; **p<0.01 vs TGFβ-stimulated. For panel B left part, *p<0.01 vs control; **p<0.05 vs control. For panel B right part, *p<0.05 vs control. Expression of deptor for these panels in parallel samples is shown in Fig. S6.

We have shown above that deptor regulates expression of collagen I (α2) mRNA (Figs. 1H and 2D). It has been reported previously that TGFβ regulates collagen I (α2) expression by a transcriptional mechanism [46]. Therefore, we used a reporter plasmid where collagen I (α2) promoter drives the luciferase gene. As expected, TGFβ increased the transcription of collagen I (α2) (Fig. 3). Expression of deptor significantly decreased the TGFβ-induced transcription of collagen I (α2) (Fig. 3A and Fig. S6A). Next, we used two independent shRNAs against deptor. Expression of either of these shRNAs increased the transcription of collagen I (α2) similar to that found with TGFβ alone (Fig. 3B and Fig. S6B). Addition of TGFβ in deptor shRNA-transfected cells did not have any further increment as compared to TGFβ alone (Fig. 3B). This could be due to the fact that TGFβ may have maximized the effect so that shDeptor could not further increase the luciferase activity in these cells. These results indicate that deptor regulates collagen I (α2) expression via a transcriptional mechanism and maintain a tonic inhibition on its gene expression in the basal state.

### Deptor regulates Hif1α in renal proximal tubular epithelial cells by mRNA translation

**Figure 4:**
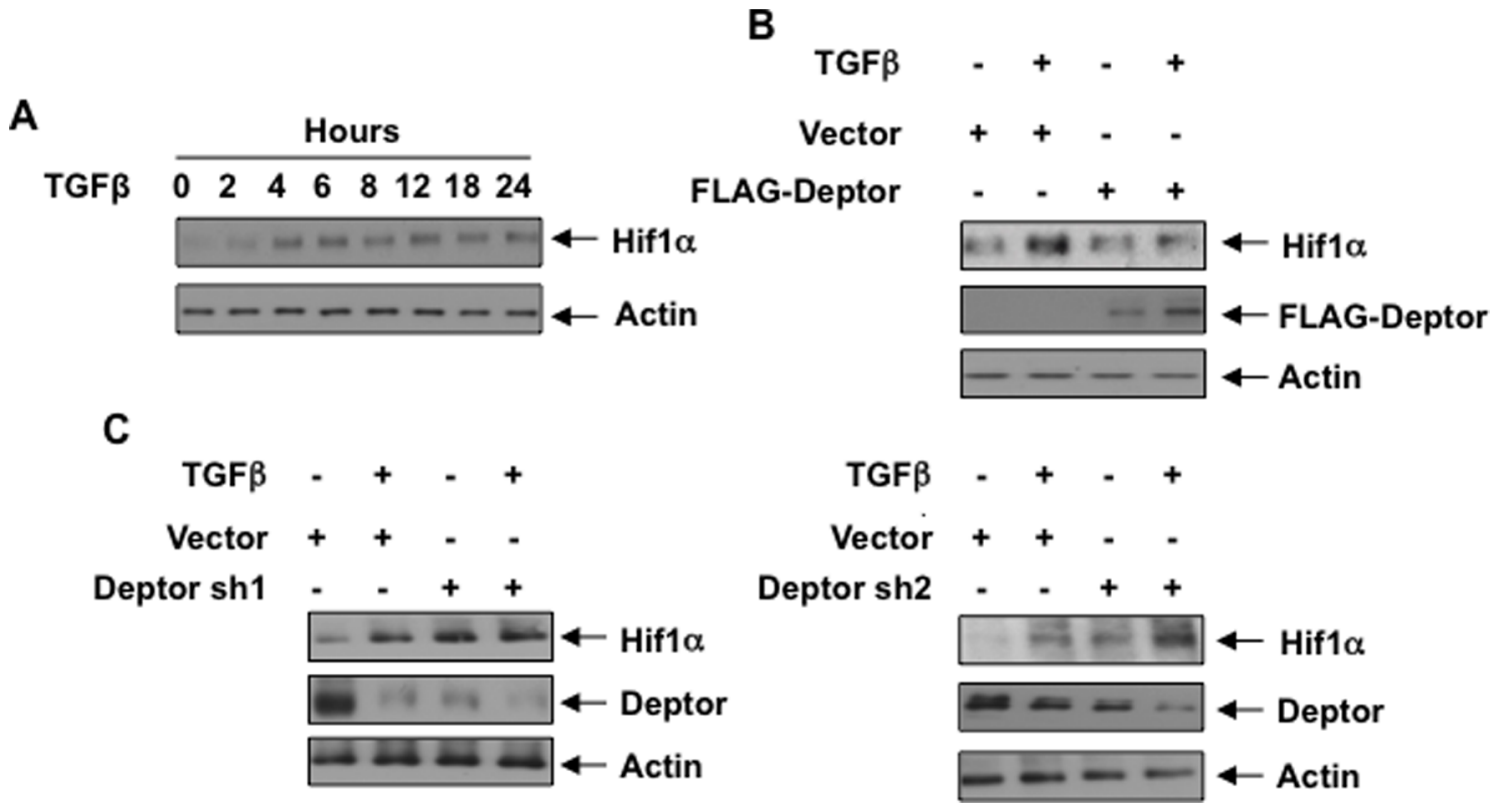
TGFβ-inhibited deptor regulates Hif1α expression. (A) Human proximal tubular epithelial cells were incubated with 2 ng/ml TGFβ for indicated period of time. The cell lysates were immunoblotted with Hif1α and actin antibodies. (B and C) Human proximal tubular epithelial cells transfected with FLAG-Deptor (panel B) or shRNAs against deptor (Deptor sh1 and Deptor sh2) were incubated with TGFβ for 24 hours (panel C). The cell lysates were immunoblotted with Hif1α, deptor, actin and FLAG antibodies as indicated. Quantifications of panel A–C are shown in Fig. S7A–S7C.

TGFβ regulates the expression of collagen I (α2) via Smad 3-dependent transcriptional activation [46], [49]. Recently, it was shown that Hif1α contributes to the Smad 3-dependent collagen I (α2) expression [50]. To systematically initiate our studies involving the mechanism of deptor regulation of collagen I (α2), we considered Hif1α as a target transcription factor. TGFβ increased the expression of Hif1α protein in human proximal tubular epithelial cells in a time-dependent manner (Fig. 4A and Fig. S7A). Interestingly, expression of deptor significantly inhibited TGFβ-induced Hif1α expression at 24 hours (Fig. 4B and Fig. S7B). To confirm this observation, we used shRNAs against deptor. Using two independent shRNAs, we found that downregulation of deptor was sufficient to increase Hif1α expression in these cells similar to TGFβ treatment (Fig. 4C and Fig. S7C). Interestingly, TGFβ did not increase Hif1α mRNA (Fig. S8) Also, deptor over expression or deptor shRNAs had no effect on Hif1α mRNA exptession (Figs. S8A and S8B).

**Figure 5:**
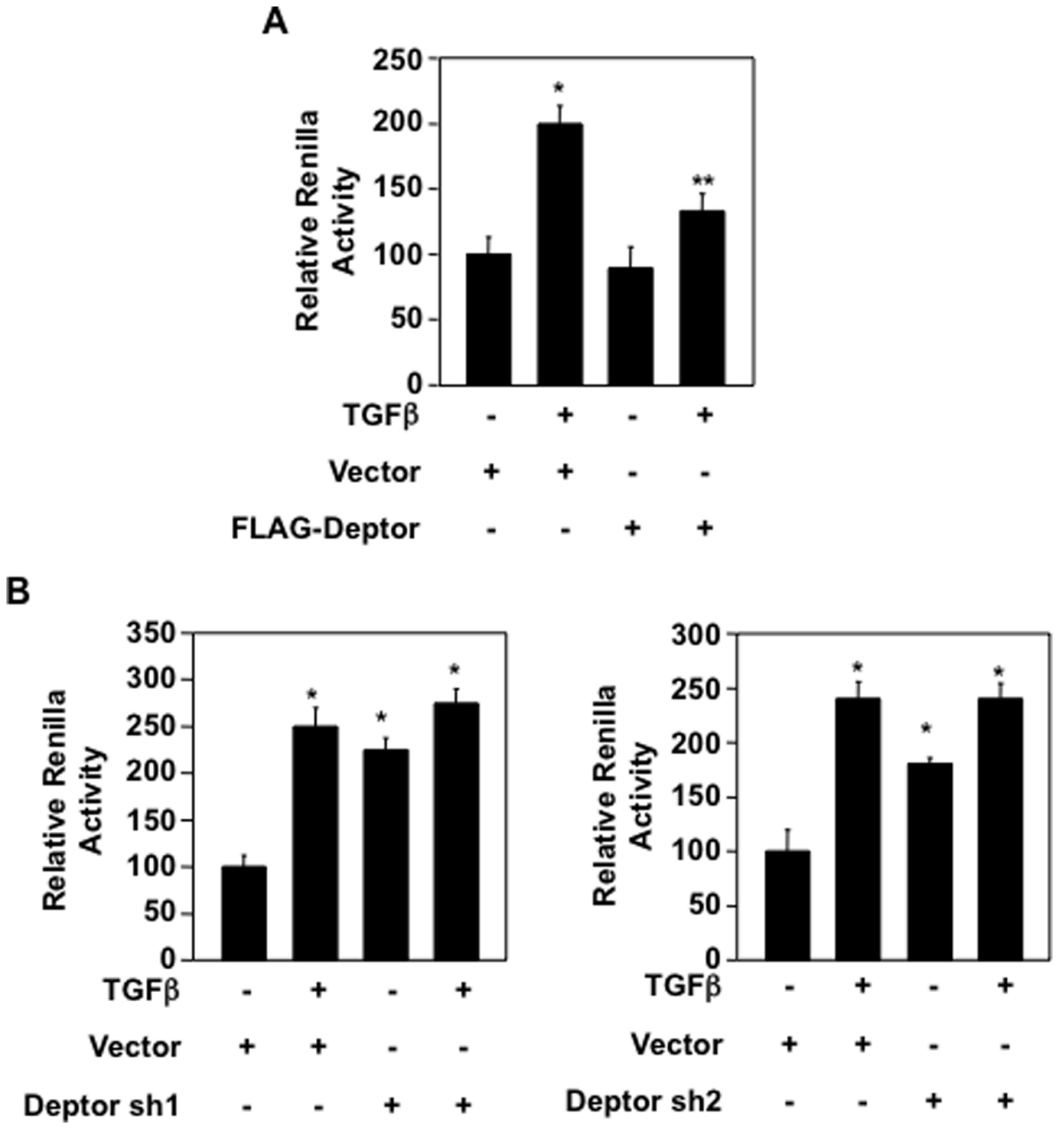
TGFβ-induced Hif1α expression is translationally regulated by deptor expression. Human proximal tubular epithelial cells were cotransfected with the Hif1α 5′UTR-fused Renilla luciferase and FLAG-Deptor (panel A) or deptor shRNAs (panel B). The transfected cells were treated with TGFβ for 24 hours. The cell lysates were used to assay Renilla luciferase activity as described [5], [39]. In panel A, Mean ± SE of 5 measurements is shown. *p<0.001 vs control; **p<0.001 vs TGFβ-stimulated. In panel B, Mean ± SE of 3 measurements is shown. *p<0.05 vs control. Expression of deptor for these panels from parallel samples is shown in Fig. S9.

TSC2 null murine embryonic fibroblasts express increased levels of Hif1α protein due to enhanced mTOR activity [51]. It was reported that this increase is due to augmented mRNA translation of Hif1α as result of the presence of 5′ terminal oligopyrimidines (5′TOP) in its untranslated region (UTR) [39], [52]. We have shown above that TGFβ-induced deptor downregulation augments the activity of both mTORC1 and mTORC2 (Figs. 1A–1D). To determine the role of deptor in regulation of Hif1α protein levels via mRNA translation, we used a reporter construct in which the 5′ UTR of Hif1α mRNA is fused to the Renilla luciferase gene (Hif1α-TOP-Lux). This reporter plasmid was transfected into proximal tubular epithelial cells. TGFβ significantly increased Hif1α-5′UTR-mdiated luciferase activity (Fig. 5). Interestingly, expression of deptor significantly inhibited the Hif1α-5′UTR-mediated reporter activity (Fig. 5A and Fig. S9A). In contrast, expression of two independent shRNAs against deptor was sufficient to increase the reporter activity similar to TGFβ treatment (Fig. 5B and Fig. S9B). Deptor shRNAs in the presence of TGFβ did not further increase the luciferase activity. These results suggest that deptor increases Hif1α protein level via increased translation of Hif1α mRNA.

### Deptor regulates Hif1α interaction with collagen I (α2) gene

**Figure 6:**
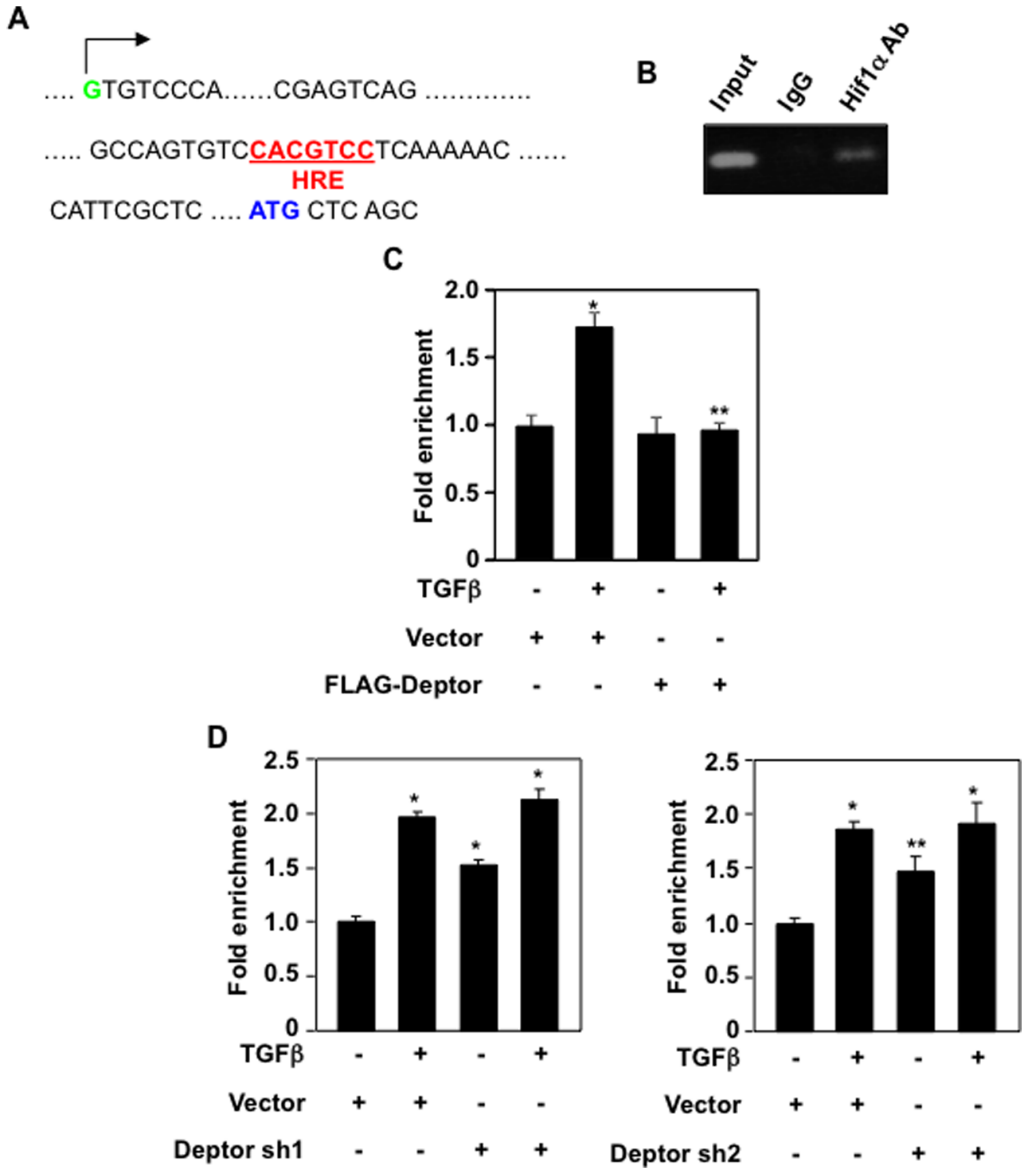
TGFβ-inhibited deptor regulates Hif1α binding to its cognate HRE in collagen I (α2) gene. (A) The sequence showing the HRE (bases denoted in red and underlined), the start codon (in blue) and the transcription initiation site (in green and indicated by arrow) of the collagen gene. (B) Chromatin immunoprecipitation assay to determine binding of Hif1α to collagen gene. Fragmented chromatins from proximal tubular epithelial cells were incubated with IgG or anti-Hif1α antibody as described in the Materials and Methods. The bound DNA was eluted and amplified with collagen gene specific primers flanking the HRE shown in panel A as described in Materials and Methods. (C and D) The cells were transfected with FLAG-deptor (panel C) or shRNAs against deptor (panel D). The transfected cells were incubated with TGFβ. Fragmented chromatin preparations were used for ChIP assay as described in panel B except the amplification was performed by real time PCR as described under Materials and Methods. Relative amount of bound Hif1α was calculated by the ratio of ChIPed DNA to input control DNA. Mean ± SE of triplicate measurements is shown. In panel C, *p<0.01vs control; **p<0.01 vs TGFβ-treated. In panel D, left panel *p<0.001 vs control. In panel D right panel, *p<0.01 vs TGFβ; **p<0.05 vs control. Expression of deptor for panel C and D is shown in parallel samples in Fig. S10.

Hif1α has been shown to regulate collagen I (α2) expression by TGFβ-stimulated Smad 3 [50]. However, analysis of the 5′ flanking sequence of collagen I (α2) gene revealed the presence of Hif1α responsive element (HRE) between the putative transcription start site and the start codon (Fig. 6A). To determine whether endogenous Hif1α occupies this site in the collagen I (α2) gene, we performed ChIP assay. As shown in Fig. 6B, we detected physical association of Hif1α with the HRE present in the collagen I (α2) 5′ flanking sequence. Next, we determined the effect of TGFβ on binding of endogenous Hif1α to this site. TGFβ significantly increased the binding of Hif1α to its cognate binding element (Figs. 6C, 6D). Interestingly, expression of deptor significantly inhibited the binding of Hif1α to the 5′ flanking sequence of collagen I (α2) gene (Fig. 6C and S10A). To confirm this effect of deptor, we used shRNAs against deptor. Two independent shRNAs significantly increased the Hif1α occupancy onto the collagen I (α2) 5′ flanking sequence similar to that with TGFβ (Fig. 6D and Fig. S10B). Addition of TGFβ to the shRNA-transfected cells did not further increase the binding of Hif1α (Fig. 6D). These results conclusively demonstrate that TGFβ-induced decrease in deptor expression results in marked recruitment of Hif1α to the collagen I (α2) gene.

### Deptor-regulated mTORC1 and not mTORC2 increases Hif1α to control collagen I (α2) expression

**Figure 7:**
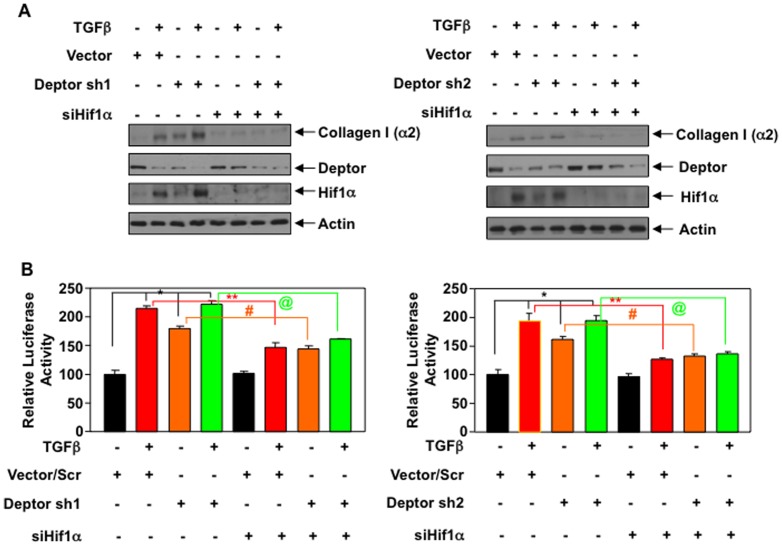
TGFβ-induced collagen expression by deptor downregulation is mediated by Hif1α. (A) Human proximal tubular epithelial cells were transfected with deptor shRNAs and siRNA against Hif1α. The transfected cells were incubated with TGFβ for 24 hours. The cell lysates were immunoblotted with collagen I (α2), deptor, Hif1α and actin antibodies as indicated. Quantifications of panel A is shown in Fig. S11A. (B) Collagen I (α2) promoter-driven luciferase reporter plasmid was co-transfected with deptor shRNAs (Deptor sh1 and Deptor sh2) and siRNA against Hif1α. The transfected cells were incubated with TGFβ for 24 hours. The cell lysates were assayed for luciferase activity as described in the Materials and Methods [5], [39]. In panel B left panel,*p<0.001 vs control; **p<0.01vs TGFβ-treated; #p<0.01 vs Deptor shRNAs alone; @p<0.001 vs shRNA against deptor plus TGFβ. In panel B right panel, *p<0.001 vs control; **p<0.05vs TGFβ-treated; #p<0.05 vs Deptor shRNAs alone; @p<0.05 vs shRNA against deptor plus TGFβ. Expression of deptor and Hif1α for panel B is shown in Fig. S711B.

Our results above suggest that downregulation of deptor by TGFβ increases Hif1α levels and that this is associated with elevated collagen I (α2) expression in proximal tubular epithelial cells. To determine the direct involvement of Hif1α in collagen I (α2) expression by deptor modulation, we transfected proximal tubular epithelial cells with deptor shRNAs along with siRNA against Hif1α. The cells were then incubated with TGFβ. As expected, TGFβ as well as shRNAs against deptor alone increased the collagen I (α2) protein levels (Figs. 7A). Interestingly, expression of siRNA against Hif1α significantly inhibited the expression of collagen I (α2) induced by TGFβ and shRNA-mediated downregulation of deptor individually as well as with TGFβ in the presence of deptor downregulation (Figs. 7A and Fig. S11A). To determine the transcriptional regulation, we used the collagen I (α2) promoter-reporter construct. Similar to the collagen I (α2) protein expression, siHif1α significantly decreased TGFβ- and Deptor shRNA-mediated collagen I (α2) transcription (Figs. 7B and Fig. S11B). Also siHif1α reduced the transcription of collagen I (α2) induced by combined action of Deptor shRNAs and TGFβ (Figs. 7B). These results conclusively demonstrate that TGFβ-induced deptor downregulation-mediated expression of collagen I (α2) utilizes Hif1α.

**Figure 8:**
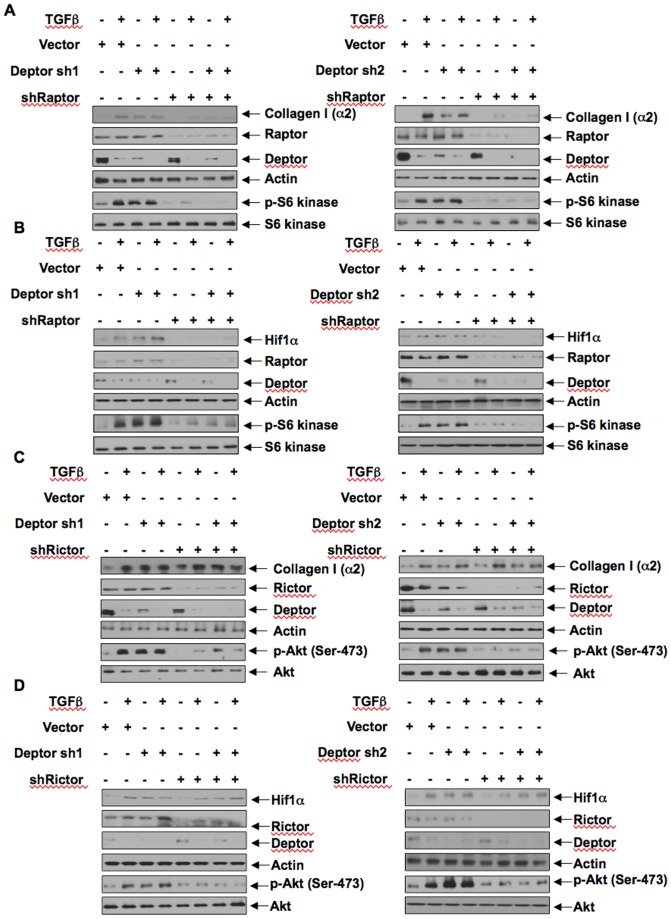
TGFβ-induced deptor downregulation uses mTORC1 and not mTORC2 to increased collagen I (α2) and Hif1α expression. (A and B) Human proximal tubular epithelial cells were transfected with deptor shRNAs (deptor sh1 and deptor sh2) along with shRNA against raptor. The transfected cells were incubated with TGFβ for 24 hours. The cell lysates were immunoblotted with collagen (α2) (panel A), Hif1α (panel B) and raptor, deptor, actin antibodies as indicated. (C and D) The cells were transfected with deptor shRNAs (Deptor sh1 and Deptor sh2) along with shRNA against rictor. The cell lysates were immunoblotted with collagen I (α2) (panel C), Hif1α (panel D) and rictor, deptor, actin antibodies as indicated. Quantifications of Fig. 8 are shown in Figs. S12A–S12D.

**Figure 9:**
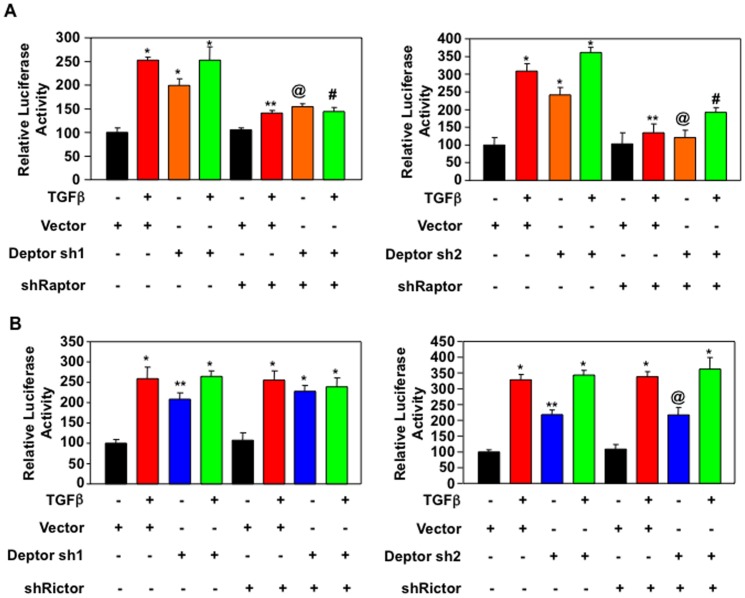
Deptor downregulation by TGFβ uses mTORC1 and not mTORC2 to increase transcription of collagen I (α2). Human proximal tubular epithelial cells were transfected with collagen I (α2) promoter-driven luciferase plasmid along with deptor shRNAs and shRNA against raptor (panel A) or shRNA for rictor (panel B). The transfected cells were incubated with TGFβ for 24 hours. The cell lysates were assayed for luciferase activity as described in the Materials and Methods [5], [39]. Mean ± SE of triplicate measurements is shown. In panel A left part, *p<0.001 vs control; **p<0.05 vs TGFβ; @p<0.05 vs shDeptor alone; #p<0.01 vs shDeptor plus TGFβ. In panel B, *p<0.01 vs control. In panel A right panel, *p<0.01 vs control; **p<0.01 vs TGFβ; @p<0.01 vs shDeptor alone; #p<0.01 vs shDeptor plus TGFβ. In panel B left panel, *p<0.01 vs control; **p<0.05 vs control. In panel B right panel, *p<0.001 vs control; **p<0.05 vs control; @p<0.01 vs control. Expression of deptor, raptor and rictor for all panels is shown in Fig. S13A and S13B.

Deptor is a component of both mTORC1 and mTORC2 [47]. We have shown above that TGFβ-induced inhibition of deptor increases activity of both these kinase complexes (Figs. 1A–1D). However, it is not known whether deptor utilizes both mTORC1 and mTORC2 to induce the expression of collagen I (α2) in response to TGFβ. To examine the contribution of mTORC1 in this process, we used shRNA against raptor, which is essential for mTORC1 activity [20], [21]. Raptor shRNA was transfected with deptor shRNAs and the cells were incubated with TGFβ. As expected, Deptor shRNA alone and along with TGFβ increased the expression of collagen I (α2) (Fig. 8A and Fig. S12A). But expression of shRaptor significantly inhibited both shDeptor- and shDeptor plus TGFβ-induced collagen I (α2) expression, which was concomitant with decrease in expression of Hif1α (Figs. 8A, 8B and Figs. S12A and S12B). To study the role of mTORC2, we used shRNA targeting rictor, a required constituent of mTORC2 activity [53]. Expression of shRictor did not have any inhibitory effect on expression of collagen I (α2) by TGFβ or shDeptor alone or in combination (Fig. 8C and Fig. S12C). Similarly shRictor did not inhibit Hif1α expression induced by TGFβ or shDeptor alone or in combination (Fig. 8D and Fig. S12D). To confirm the role of mTORC1 in collagen I (α2) expression, we used the reporter construct with collagen I (α2) promoter. Downregulation of raptor inhibited the transcription of collagen I (α2) stimulated by TGFβ, shDeptor and shDeptor with TGFβ (Fig. 9A and S13A). In contrast, inhibition of rictor did not have any effect on collagen I (α2) transcription (Fig. 9B and Fig. S13B). These results indicate a preferential use of mTORC1 over mTORC2 downstream of deptor downregulation by TGFβ to increase collagen I (α2) expression.

## Discussion

**Figure 10:**
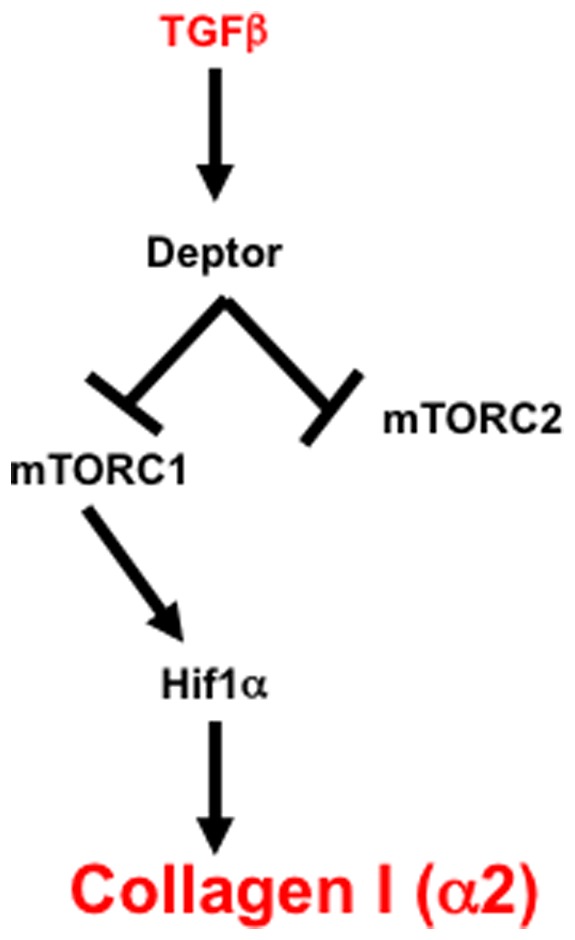
Cartoon summarizes the results demonstrating the involvement of deptor and mTORC1 in Hif1α expression for collagen I (α2) expression in response to TGFβ.

In this report, we provide the first evidence that TGFβ-induced deptor downregulation contributes to fibrotic gene collagen I (α2) expression by a transcriptional mechanism. Our results demonstrate that deptor downregulation by TGFβ increases Hif1α translation to increase its protein level, which subsequently binds to its cognate HRE in the collagen I (α2) gene to increase its transcription. Finally, we show that TGFβ-stimulated mTORC1 and not mTORC2 downstream of deptor downregulation contributes to increased expression of Hif1α and collagen I (α2) (Fig. 10).

Canonical TGFβ-stimulated Smad 3 signaling has been shown to regulate fibrotic gene expression [54]. However, cross-talk between the noncanonical PI 3 kinase/Akt signaling and Smad 3 is required for expression of two matrix proteins fibronectin and collagen I (α2) [13], [46]. Recently, we and others have shown that in diabetic kidney disease in which TGFβ plays a significant role to produce extracellular matrix, administration of rapamycin, which inhibits both mTORC1 and mTORC2 in mice, resulted in marked reduction in matrix proteins including collagen I (α2) [33], [34], [55], [56]. Rapamycin also inhibits basal and TGFβ-induced expression of collagen I (α2) in renal glomerular mesangial cells and proximal tubular epithelial cells (Fig. S1) [17]. Homozygous deletion of mTOR in mice is embryonically lethal indicating its importance in normal physiology [57]. PI 3 kinase/mTOR activity is essential for normal physiological function of renal cells [58]. When patients with chronic allograft nephropathy were changed from calcineurin to rapamycin treatment, 62% of them showed new onset of proteinuria [59]. In fact 36% showed nephritic level proteinuria. In another study, one third of the patients showed *de novo* 1 g/day proteinuria when switched to rapamycin [60]. Also, renal transplant patients treated with rapamycin show increased proteinuria due to renal damage including tubular damage [61]. In animals with puromycin aminonucleoside-induced nephrotoxicity, treatment with rapamycin produced loss of renal function [62]. More recently using renal podocyte-specific raptor knockout mice, Godel et al have reported severe proteinuria at early stage [63]. These results indicate that complete loss of mTOR is detrimental to the normal homeostasis of renal cells.

Deptor was identified as an mTOR interacting protein [47]. The C-terminal PDZ segment of deptor interacts with the FAT domain of mTOR and prevents the kinase activity of mTOR present in both mTORC1 and mTORC2 [47]. Thus deptor represents a natural inhibitor, which maintains the basal activity of both kinase complexes. Increased mTOR kinase activity represents a major pathology in many cancers [21]. It was shown that the level of deptor is significantly low in many cancers [47], [64]. In fact, deptor inhibition was the sole cause for resistance of cancer cells to apoptosis [47]. Sustained activation of mTOR is seen in fibrotic renal diseases such as diabetic nephropathy in which TGFβ plays an important role in developing fibrosis [30], [31], [32], [33], [34], [65]. Interestingly, TGFβ inhibits deptor expression with concomitant increase in both mTORC1 and mTORC2 activities (Fig. 1A–1D). Moreover, suppression of deptor by prolonged incubation with TGFβ contributes to the expression of collagen I (α2) by a transcriptional mechanism (Figs. 1–3).

Although deptor inhibits the activity of both mTORC1 and mTORC2, two other proteins, tuberin and PRAS40, negatively regulate the activity of the mTORC1 [66], [67]. Inactivation of PRAS40 and tuberin by Akt-mediated phosphorylation results in increased mTORC1 activity. We have shown recently that rapid activation of mTORC1 in renal cells involves phosphorylation/inactivation of these two proteins [16], [38], [42], [68]. However, expression of deptor did not have any effect on phosphorylation of tuberin and PRAS40 when the proximal tubular epithelial cells were incubated with TGFβ for 15 minutes (rapid activation) (Figs. S14A and S14B). Consequently, deptor did not inhibit TGFβ-stimulated early mTORC1 activation as indicated by phosphorylation of S6 kinase and 4EBP-1 (Figs. S15A and S15B). Similarly, expression of deptor had no effect on TGFβ-induced phosphorylation of Akt at Ser-473, indicator of mTORC2 activation (Fig. S16). In contrast to these results we found significant inhibition of prolonged activation of both mTORC1 and mTORC2 by deptor, which results in attenuation of collagen I (α2) expression (Figs. 1E–1I). Mechanistically, activation of mTORC1 involves phosphorylation of both PRAS40 and tuberin. In fact, we found that expression of deptor blocked phosphorylation of both PRAS40 and tuberin when the cells were incubated with TGFβ for prolonged period of time (Figs. S17A and S17B). These results indicate that TGFβ induces a deptor-independent rapid activation of mTOR; however, expression of collagen I (α2) requires deptor-mediated activation of mTOR induced by prolonged TGFβ treatment (Fig. 1H, 1I, Fig. 2D, 2E and Fig. 3).

The transcription factor Hif1 is a heterodimer of Hif1α and Hif1β. This complex formation is regulated by the availability of Hif1α subunit, which is sensitive to normoxia and undergoes degradation by the proline hydroxylase domain proteins [69]. Level of Hif1α is significantly elevated by hypoxia, which undergoes phosphorylation by ATM to increase REDD1 that activates the tuberous sclerosis complex and results in inhibition of mTORC1 activity [70], [71], [72]. In addition to hypoxia, oncogenes, mutations in metabolic enzyme genes and tumor suppressor genes can cause upregulation of Hif1α protein [73], [74], [75]. Also, increased Hif1α level is present in cells with activated mTORC1 due to mutation in TSC1 or TSC2 which removes negative regulatory constraint on Rheb-GTP necessary for mTORC1 activation [20], [21], [51]. More recently, analysis of genome sequence of 750 cancer samples including renal cancer identified several point mutations in the C-terminus of mTOR. Two of these point mutants showed constitutive mTORC1 activity without any increase in mTORC2 activity [76], [77]. All these modes of mTORC1 activation result in increased Hif1a levels due to enhanced 5′TOP mRNA translation of Hif1α [36], [51], [78]. However, a recent study revealed a role of mTORC2 in Hif1α expression [79]. In the present study, downregulation of deptor by TGFβ, which increases both mTORC1 and mTORC2 activities, increased the levels of Hif1α in a prolonged manner (Fig. 4A). Also, our results for the first time demonstrate that deptor regulates TGFβ-induced expression of Hif1α (Fig. 4B–4D). The deptor-regulated increase in Hif1α is the result of increased translation of 5′TOP containing Hif1α mRNA (Fig. 5).

The role of Hif1α in cancer is extensively studied, where upregulation of all 13 glycolytic genes to exert Warburg effect is under the influence of this transcription factor [80]. In addition, Hif1α supports angiogenesis by increasing the expression of VEGF under hypoxic and normoxic conditions [51], [69], [80]. Also, we have shown that in the hamartoma syndrome tuberous sclerosis, normoxic elevation of mTOR activity enhances the PTEN tumor suppressor gene expression via upregulation of Hif1α [39], [41]. Furthermore, Hif1α has been implicated in the pathogenesis of atherosclerosis [81]. Hif1α can physically interact with various transcription factors to increase the expression of the target genes. In fact, Hif1α has been shown to physically interact with the TGFβ-specific Smad3 transcription factor to increase expression of VEGF, collagen I (α2) and endoglin [50], [80], [82], [83]. Interestingly, we identified a Hif1α responsive element in the collagen I (α2) gene between the transcription initiation site and start codon (Fig. 6A). For the first time, we show that Hif1α directly binds to this site in proximal tubular epithelial cells (Fig. 6B). Furthermore, we provide evidence for a direct role of deptor in mediating Hif1α binding to this site (Figs. 6C and 6D). In fact, we demonstrate that deptor-regulated expression of collagen I (α2) protein is indeed mediated by Hif1α-dependent transcription (Figs. 7A and 7B).

As described above, deptor constitutively binds to mTOR; consequently it is present in both mTORC1 and mTORC2 [47]. Interestingly, it was shown previously that when overexpressed, deptor inhibited only mTORC1 and increased mTORC2 activity, which is necessary for maintenance of certain cancers such as multiple myeloma [47]. In contrast to these results, in the present study when deptor was overexpressed in proximal tubular epithelial cells, it inhibited mTORC2 activity induced by TGFβ (Fig. 1G). Thus our results demonstrate that deptor regulates both mTORC1 and mTORC2 activities in proximal tubular epithelial cells (Figs. 1E, 1F and 1G). We also show that deptor controls the expression of collagen I (α2) gene in response to TGFβ by a transcriptional mechanism (Figs. 2 and 3). Importantly, when we specifically inhibited mTORC1 activity the increase in collagen I (α2) protein expression and its transcription by deptor downregulation or TGFβ alone or in combination was significantly inhibited (Figs. 8A and 9A). Furthermore, inhibition of mTORC1 alone blocked TGFβ- and shDeptor-induced Hif1α protein levels (Fig. 8B). Interestingly, when mTORC2 activity was inhibited by rictor downregulation, there was no effect of TGFβ-induced suppression of deptor on collagen I (α2) protein expression and transcription (Figs. 8C and 9B). Also, Hif1α expression was unaffected (Fig. 8D). These results conclusively suggest that mTORC2, although activated by TGFβ-mediated downregulation of deptor, acts as a bystander and does not contribute to the expression of collagen I (α2). Use of rapamycin to inhibit mTORC1 produces adverse side effects in the kidney [59], [60], [61], [62]. Many other direct mTOR kinase inhibitors are being developed; however, they display severe toxicity. Since decrease in deptor contributes to the pathologic action of TGFβ to increase expression of tubular collagen I (α2), development of safe compounds that increase the levels of deptor, resulting in inhibition of mTORC1, may be beneficial for fibrotic renal diseases.

## Supporting Information

Figure S1 **Rapamycin inhibits TGFβ-induced collagen I (α2) expression in human proximal tubular epithelial cells.** The cells were treated with 25 nM rapamycin for 1 hour prior to incubation with 2 ng/ml TGFβ for 24 hours. The cell lysates were immunoblotted with collagen I (α2) and actin antibodies.
(PDF)

Figure S2 **Quantification of the results shown in** **Figs. 1A–1G** **.** (A) Ratio of deptor to actin. Mean ± SE of 3 independent experiments is shown. *p<0.01 vs 0 hour. (B) Ratio of phospho-S6 kinase to S6 kinase. Mean ± SE of 3 independent experiments is shown. *p<0.01 vs 0 hour. (C) Ratio of phospho-4EBP-1 to 4EBP-1. Mean ± SE of 3 independent experiments is shown. *p<0.001 vs 0 hour. (D) Ratio of phospho-Akt (Ser-473) (left panel) and phospho-Akt (Thr-308) (right panel) to Akt. Mean ± SE of 3 independent experiments is shown. *p<0.001 vs 0 hour. (E) Ratio of phospho-S6 kinase to S6 kinase. Mean ± SE of 5 independent experiments is shown. *p<0.001 vs vector; **p<0.01 vs TGFβ-stimulated. (F) Ratio of phospho-4EBP-1 to 4EBP-1. Mean ± SE of 5 independent experiments is shown. *p<0.001 vs vector; **p<0.01 vs TGFβ-treated. (G) Ratio of phospho-Akt (Ser-473) (left panel) and phospho-Akt (Thr-308) (right panel) to Akt. Mean ± SE of 4 independent experiments is shown. *p<0.001 vs vector; **p<0.01 vs TGFβ-treated.
(PDF)

Figure S3 **Expression of deptor for the results shown in** **Figure 1H** **, (A).** Human proximal tubular epithelial cells were transfected with FLAG-Deptor expression vector prior to incubation with 2 ng/ml TGFβ as described in the legend of Fig. 1H. The cell lysates were immunoblotted with FLAG and actin antibodies. (B) Quantification of the results shown in Fig. 1I. Ratio of collagen I (α2) to actin. Mean ± SE of 4 independent experiments is shown. *p<0.01 vs vector; **p<0.01 vs TGFβ-treated.
(PDF)

Figure S4 **Quantification of the results shown in** **Figs. 2A–2C** **.** (A) Ratio of phospho-S6 kinase to S6 kinase. Mean ± SE of 4 independent experiments is shown. *p<0.001 vs vector. (B) Ratio of phospho-4EBP-1 to 4EBP-1 is shown. Means ± SE of 5 for left and 4 experiments for right panels respectively are shown. *p<0.001 vs vector. (C) Ratio of phospho-Akt to Akt is shown. Means ± SE of 4 for left and 5 experiments for right panels respectively are shown. *p<0.001 vs vector.
(PDF)

Figure S5 **Expression of deptor for the results shown in** **Figure 2D** **, (A).** Human proximal tubular epithelial cells were transfected with expression vectors containing shRNAs against deptor (Deptor sh1 and Deptor sh2) prior to incubation with 2 ng/ml TGFβ as described in the legend of Fig.2D. The cell lysates were immunoblotted with deptor and actin antibodies. (B) Quantification of the results shown in Fig. 2E. Ratio of collagen I (α2) to actin is shown. Means ± SE of 4 independent experiments are shown. *p<0.001 vs vector alone. (C) Rescue of deptor downregulation by human deptor expression in mouse proximal tubular epithelial cells to show specificity of deptor shRNA. Mouse proximal tubular epithelial cells were transfected with shRNA against mouse deptor along with FLAG-tagged human deptor expression vector as indicated. The cells were incubated with TGFβ for 24 hours. Expression of collagen I (α2), endogenous deptor, FLAG-tagged human deptor and actin are shown.
(PDF)

Figure S6 **Expression of deptor for the results shown in** **Figure 3** **.** Human proximal tubular epithelial cells were transfected with expression vectors containing FLAG-Deptor (Panel A) or shRNAs against deptor (Panel B) prior to incubation with TGFβ as described in the legend of Fig. 3. The cell lysates were immunoblotted with FLAG and actin antibodies (Panel A) and deptor and actin antibodies (Panel B).
(PDF)

Figure S7 **Quantification of the results shown in** **Fig. 4** **.** (A) Ratio of Hif1α to actin. Mean ± SE of 3 independent experiments is shown. For increase in 2 hours, *p<0.05 vs 0 hour; for increase in 4–24 hours *p<0.01 vs 0 hour. (B) Ratio of Hif1α to actin. Mean ± SE of 4 independent experiments is shown. *p<0.001 vs vector; **p<0.001 vs TGFβ-treated. (C) Ratio of Hif1α to actin. Mean ± SE of 4 independent experiments is shown. *p<0.05 vs vector alone for left panel; *p<0.001 vs vector for the right panel.
(PDF)

Figure S8 **TGFβ does not regulate Hif1α mRNA expression.** Human proximal tubular epithelial cells were transfected with FLAG-tagged Deptor expression vector (panel A) or Deptor sh1 or sh2 (panel B) as indicated followed by incubation with 2 ng/ml TGFβ for 24 hours. Expression of Hif1α mRNA was determined by real time RT-PCR as described in the Materials and Methods. Mean ± SE of triplicate measurements is shown. Bottom panels show FLAG-tagged deptor (panel A), deptor (panel B) and actin expression in parallel samples.
(PDF)

Figure S9 **Expression of deptor for the results shown in** **Figure 5** **.** Human proximal tubular epithelial cells were transfected with expression vectors containing FLAG-Deptor (Panel A) or shRNAs against deptor (Panel B) prior to incubation with TGFβ as described in the legend of Fig. 5. The cell lysates were immunoblotted with FLAG and actin antibodies (Panel A) and deptor and actin antibodies (Panel B).
(PDF)

Figure S10 **Expression of deptor for the results shown in** **Figure 6C and 6D** **.** Human proximal tubular epithelial cells were transfected with expression vectors containing FLAG-Deptor (Panel A) as described in Fig. 6C or shRNAs against deptor (Panel B) as described in Fig. 6D prior to incubation with TGFβ. The cell lysates were immunoblotted with FLAG and actin antibodies (Panel A) and deptor and actin antibodies (Panel B).
(PDF)

Figure S11 **Quantification of the results shown in** **Fig. 7A** **.** (A) Ratio of collagen I (α2) to actin. Mean ± SE of 4 independent experiments is shown. *p<0.001 vs vector alone. **p, @p, #p<0.001 vs TGFβ, shDeptor and shDeptor plus TGFβ, respectively. (B) Expression of deptor and Hif1α for the results shown in Figure 7B. Human proximal tubular epithelial cells were transfected with vector or scramble RNA (Scr) or shRNAs against deptor (Deptor sh1 and Deptor sh2) along with siRNA against Hif1α prior to incubation with TGFβ as described in the legend of Fig.7B. The cell lysates were immunoblotted with deptor, Hif1α and actin antibodies.
(PDF)

Figure S12 **Quantification of the results shown in** **Fig. 8** **.** Ratios of collagen I (α2) to actin for Fig. 8A and 8C (panels A and C) and ratio of Hif1α to actin for Fig. 8B and 8D (panels B and D) are shown. Means ± SE of 4 independent experiments are shown for A–C and for left panel of D. For panel D right panel, mean ± SE of 5 experiments is shown. For panels A and B, *p<0.001 vs vector alone. **p, @p, #p<0.001 vs TGFβ, shDeptor and shDeptor plus TGFβ, respectively. For panels C and D, *p<0.001 vs vector alone.
(PDF)

Figure S13 **Expression of raptor, rictor, deptor, and activation of mTORC1 (phospho-S6 kinase) and activation of mTORC2 (phosphorylation of Akt at Ser-473) for the results shown in** **Figure 9** **.** Human proximal tubular epithelial cells were transfected with vector or deptor shRNA expression plasmids along with raptor shRNA (Panel A) or rictor shRNA (Panel B) prior to incubation with TGFβ as described in the legend of Fig. 9. The cell lysates were immunoblotted against raptor, phospho-S6 kinase (Thr-389), S6 kinase (panel A), rictor, phospho-Akt (Ser-473), Akt (Panel B), deptor and actin antibodies as indicated.
(PDF)

Figure S14 **Expression of deptor does not inhibit rapid phosphorylation of Akt substrates PRAS40 and tuberin in response to TGFβ.** Human proximal tubular epithelial cells were transfected with vector or FLAG-Deptor. The transfected cells were incubated with 2 ng/ml TGFβ for 15 minutes. The cell lysates were immunoblotted with phospho-PRAS40 (Thr-246), PRAS40 (Panel A) and phospho-tuberin (Thr-1462), tuberin (Panel B) antibodies. Expression of deptor was detected by FLAG immunoblot.
(PDF)

Figure S15 **Expression of deptor does not inhibit rapid activation of mTORC1 in response to TGFβ.** Human proximal tubular epithelial cells were transfected with vector or FLAG-Deptor. The transfected cells were incubated with 2 ng/ml TGFβ for 15 minutes. The cell lysates were immunoblotted with antibodies for phospho-S6 kinase (Thr-389) (panel A) and phospho-4EBP-1 (Thr-37/46) (panel B) as indicators of mTORC1 activation. The lysates were also immunoblotted with FLAG antibody and S6 kinase (Panel A) and 4EBP-1 (Panel B) antibodies.
(PDF)

Figure S16 **Expression of deptor does not inhibit rapid activation of mTORC2.** Human proximal tubular epithelial cells were transfected with vector or FLAG-Deptor. The transfected cells were incubated with 2 ng/ml TGFβ for 15 minutes. The cell lysates were immunoblotted with phospho-Akt (Ser-473) antibody as indicator of mTORC2 activation. The lysates were also immunoblotted with FLAG and Akt antibodies.
(PDF)

Figure S17 **Expression of deptor inhibits phosphorylation of Akt substrates PRAS40 and tuberin in response to prolonged TGFβ incubation.** Human proximal tubular epithelial cells were transfected with vector or FLAG-Deptor. The transfected cells were incubated with 2 ng/ml TGFβ for 24 hours. The cell lysates were immunoblotted with phospho-PRAS40 (Thr-246), PRAS40 (Panel A) and phospho-tuberin (Thr-1462), tuberin (Panel B) antibodies. Expression of deptor was detected by FLAG immunoblot.
(PDF)

Table S1 **List of antibodies used in this study.**
(PDF)
