# Supplementary figures and images for: TGFβ-Induced Deptor Suppression Recruits mTORC1 and Not mTORC2 to Enhance Collagen I (α2) Gene Expression
Source: PLoS One. 2014 Oct 15;9(10):e109608. doi: 10.1371/journal.pone.0109608 (PMC4198127; doi:10.1371/journal.pone.0109608)

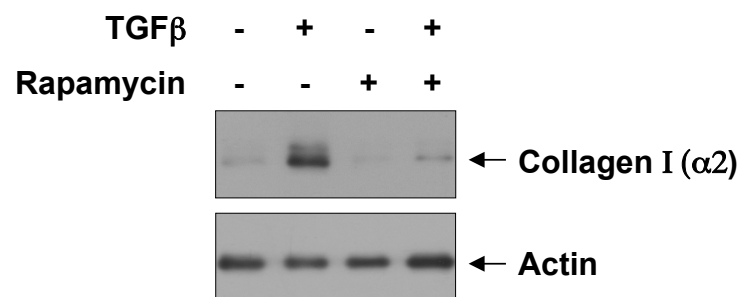

Supplement: Figure S1 — Rapamycin inhibits TGFβ-induced collagen I (α2) expression in human proximal tubular epithelial cells. The cells were treated with 25 nM rapamycin for 1 hour prior to incubation with 2 ng/ml TGFβ for 24 hours. The cell lysates were immunoblotted with collagen I (α2) and actin antibodies. (PDF) [file pone.0109608.s001.pdf]

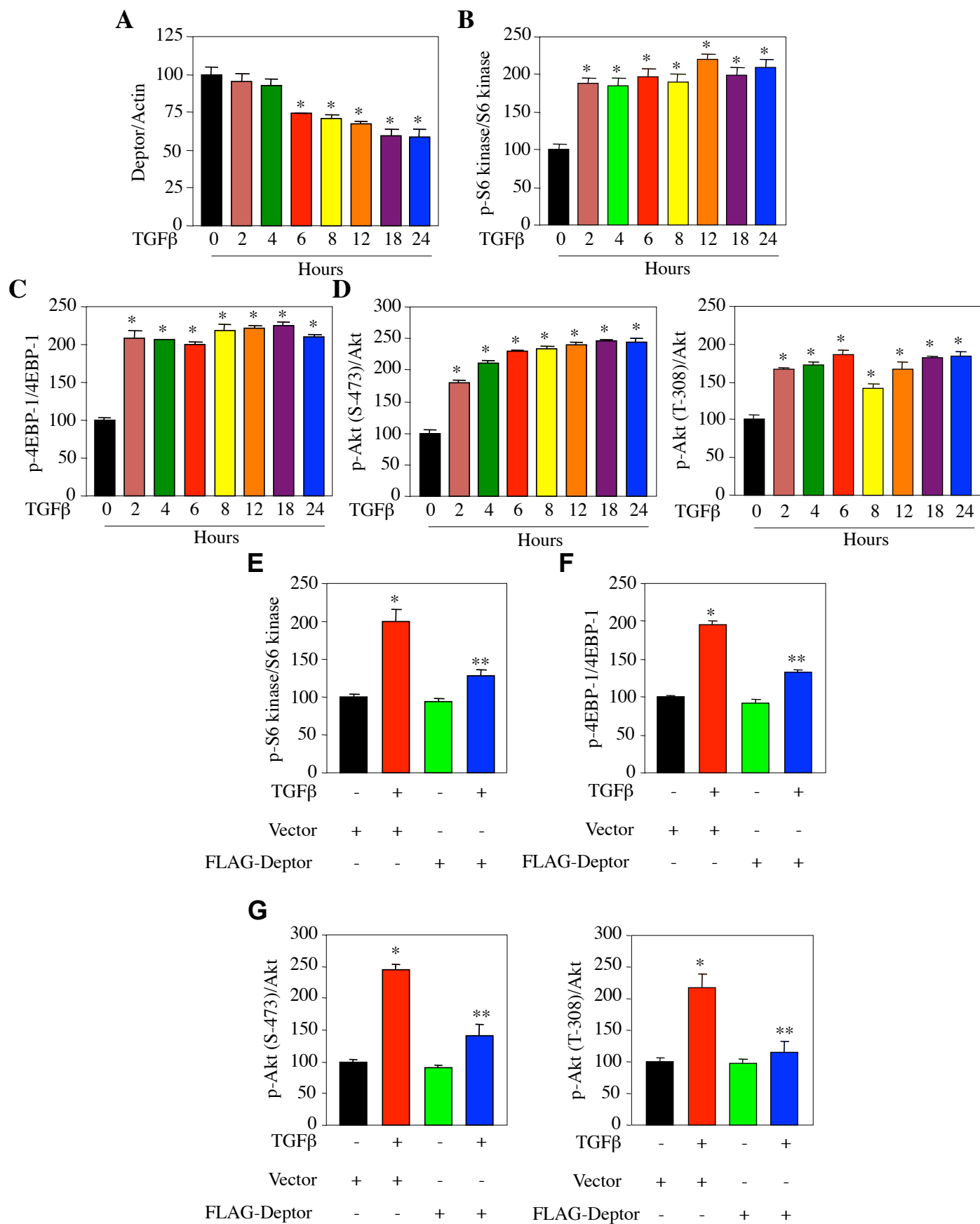

Supplementary Fig. S2  
Das F. et al

Supplement: Figure S2 — Quantification of the results shown in Figs. 1A–1G . (A) Ratio of deptor to actin. Mean ± SE of 3 independent experiments is shown. *p<0.01 vs 0 hour. (B) Ratio of phospho-S6 kinase to S6 kinase. Mean ± SE of 3 independent experiments is shown. *p<0.01 vs 0 hour. (C) Ratio of phospho-4EBP-1 to 4EBP-1. Mean ± SE of 3 independent experiments is shown. *p<0.001 vs 0 hour. (D) Ratio of phospho-Akt (Ser-473) (left panel) and phospho-Akt (Thr-308) (right panel) to Akt. Mean ± SE of 3 independent experiments is shown. *p<0.001 vs 0 hour. (E) Ratio of phospho-S6 kinase to S6 kinase. Mean ± SE of 5 independent experiments is shown. *p<0.001 vs vector; **p<0.01 vs TGFβ-stimulated. (F) Ratio of phospho-4EBP-1 to 4EBP-1. Mean ± SE of 5 independent experiments is shown. *p<0.001 vs vector; **p<0.01 vs TGFβ-treated. (G) Ratio of phospho-Akt (Ser-473) (left panel) and phospho-Akt (Thr-308) (right panel) to Akt. Mean ± SE of 4 independent experiments is shown. *p<0.001 vs vector; **p<0.01 vs TGFβ-treated. (PDF) [file pone.0109608.s002.pdf]

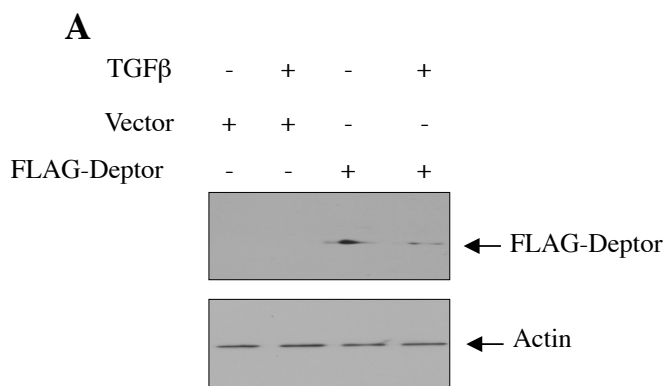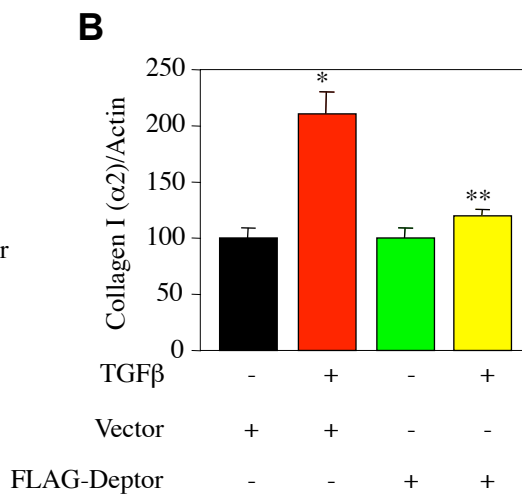

Supplement: Figure S3 — Expression of deptor for the results shown in Figure 1H , (A). Human proximal tubular epithelial cells were transfected with FLAG-Deptor expression vector prior to incubation with 2 ng/ml TGFβ as described in the legend of Fig. 1H. The cell lysates were immunoblotted with FLAG and actin antibodies. (B) Quantification of the results shown in Fig. 1I. Ratio of collagen I (α2) to actin. Mean ± SE of 4 independent experiments is shown. *p<0.01 vs vector; **p<0.01 vs TGFβ-treated. (PDF) [file pone.0109608.s003.pdf]

**A**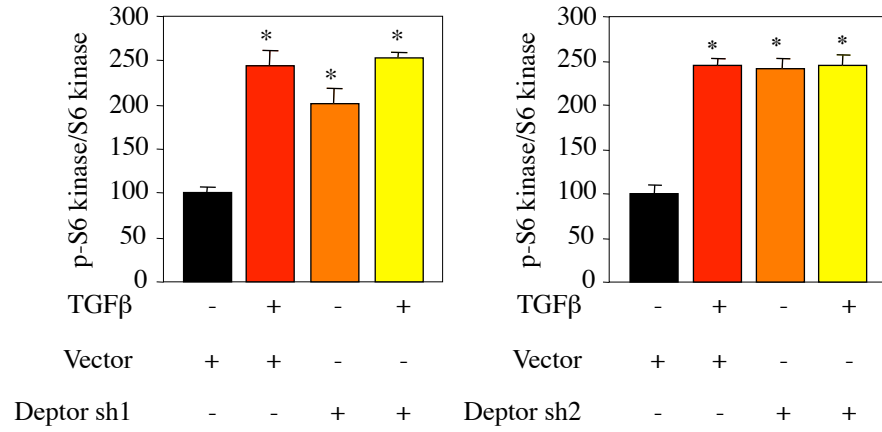**B**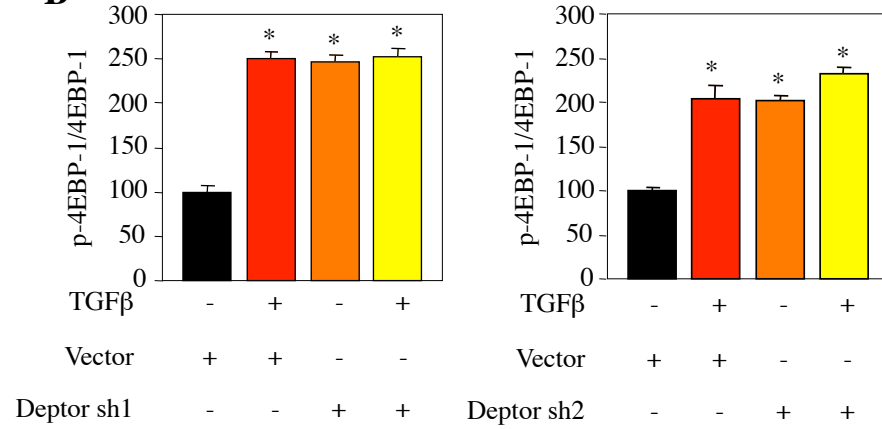**C**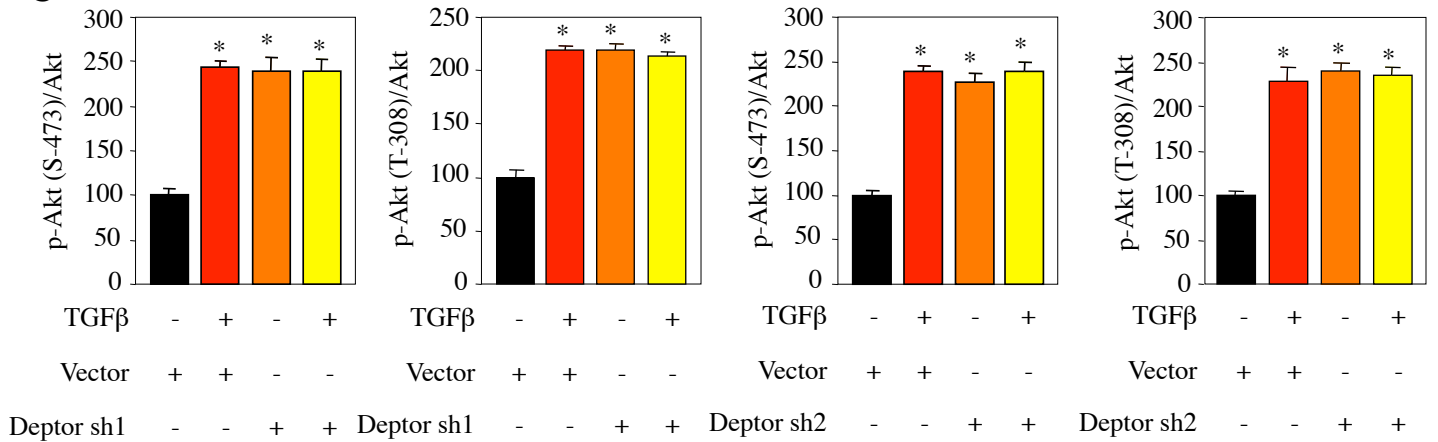

Left Panel

RightPanel

Supplement: Figure S4 — Quantification of the results shown in Figs. 2A–2C . (A) Ratio of phospho-S6 kinase to S6 kinase. Mean ± SE of 4 independent experiments is shown. *p<0.001 vs vector. (B) Ratio of phospho-4EBP-1 to 4EBP-1 is shown. Means ± SE of 5 for left and 4 experiments for right panels respectively are shown. *p<0.001 vs vector. (C) Ratio of phospho-Akt to Akt is shown. Means ± SE of 4 for left and 5 experiments for right panels respectively are shown. *p<0.001 vs vector. (PDF) [file pone.0109608.s004.pdf]

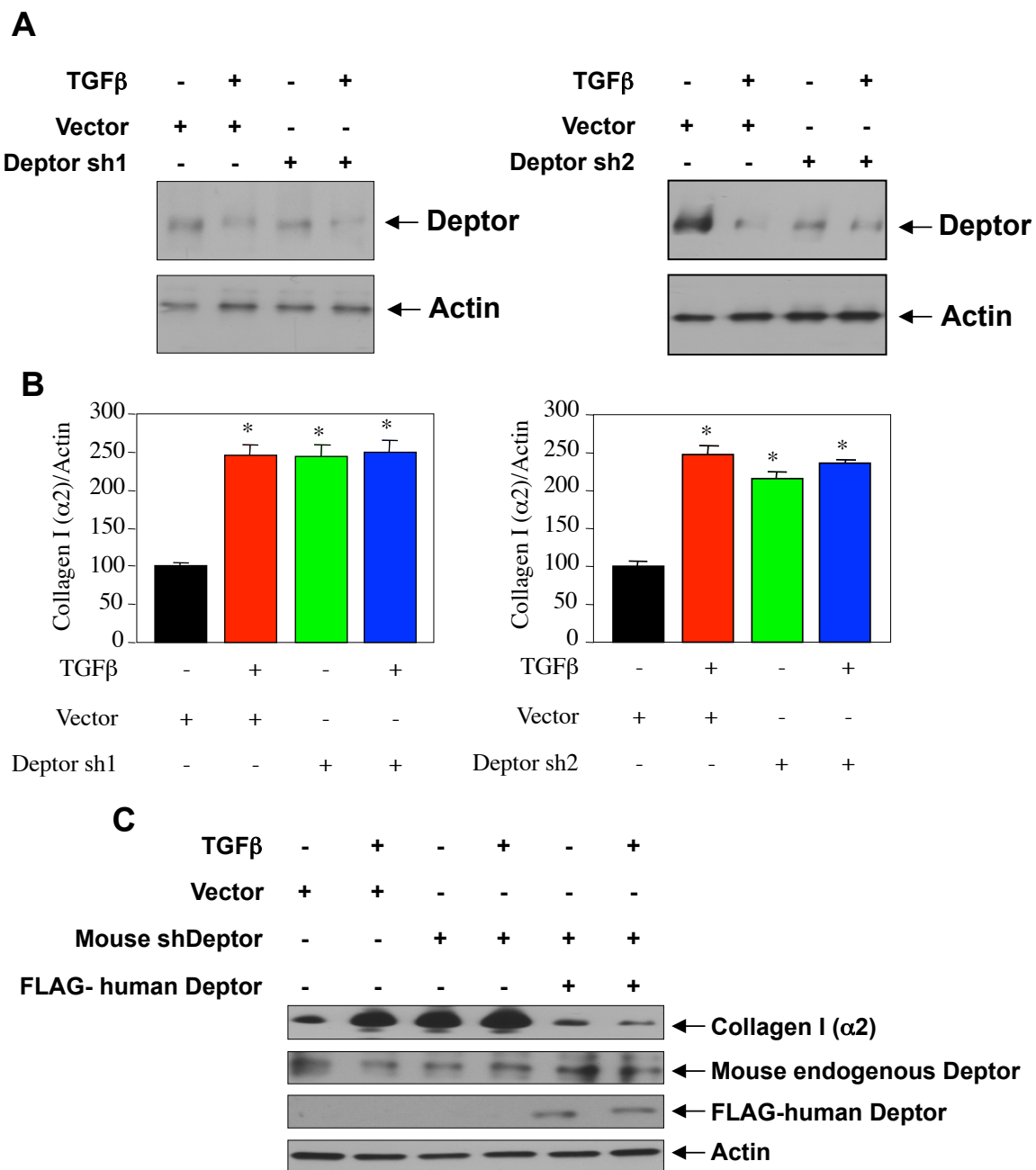

Supplement: Figure S5 — Expression of deptor for the results shown in Figure 2D , (A). Human proximal tubular epithelial cells were transfected with expression vectors containing shRNAs against deptor (Deptor sh1 and Deptor sh2) prior to incubation with 2 ng/ml TGFβ as described in the legend of Fig.2D. The cell lysates were immunoblotted with deptor and actin antibodies. (B) Quantification of the results shown in Fig. 2E. Ratio of collagen I (α2) to actin is shown. Means ± SE of 4 independent experiments are shown. *p<0.001 vs vector alone. (C) Rescue of deptor downregulation by human deptor expression in mouse proximal tubular epithelial cells to show specificity of deptor shRNA. Mouse proximal tubular epithelial cells were transfected with shRNA against mouse deptor along with FLAG-tagged human deptor expression vector as indicated. The cells were incubated with TGFβ for 24 hours. Expression of collagen I (α2), endogenous deptor, FLAG-tagged human deptor and actin are shown. (PDF) [file pone.0109608.s005.pdf]

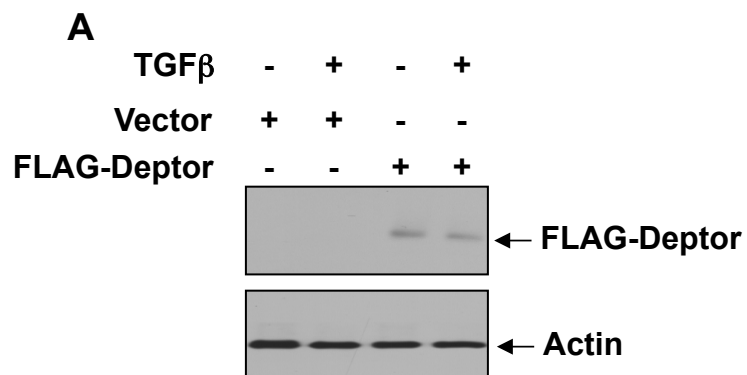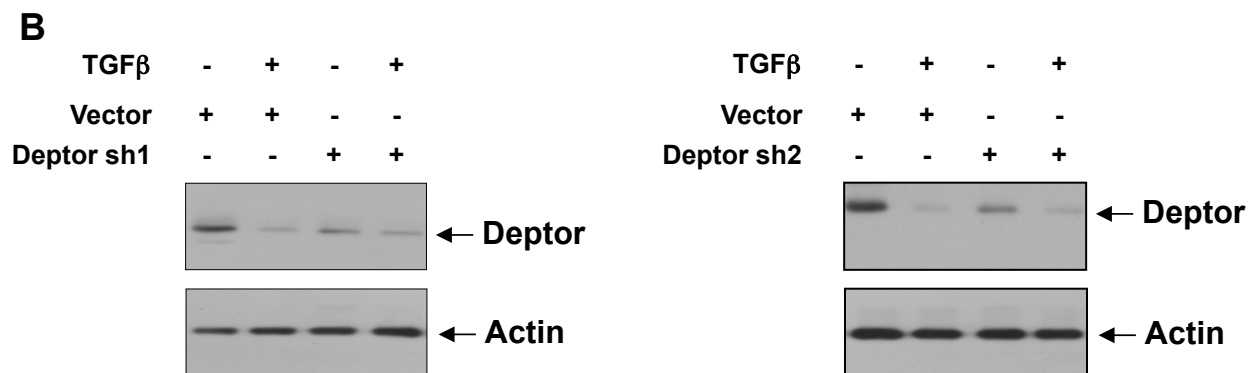

Supplement: Figure S6 — Expression of deptor for the results shown in Figure 3 . Human proximal tubular epithelial cells were transfected with expression vectors containing FLAG-Deptor (Panel A) or shRNAs against deptor (Panel B) prior to incubation with TGFβ as described in the legend of Fig. 3. The cell lysates were immunoblotted with FLAG and actin antibodies (Panel A) and deptor and actin antibodies (Panel B). (PDF) [file pone.0109608.s006.pdf]

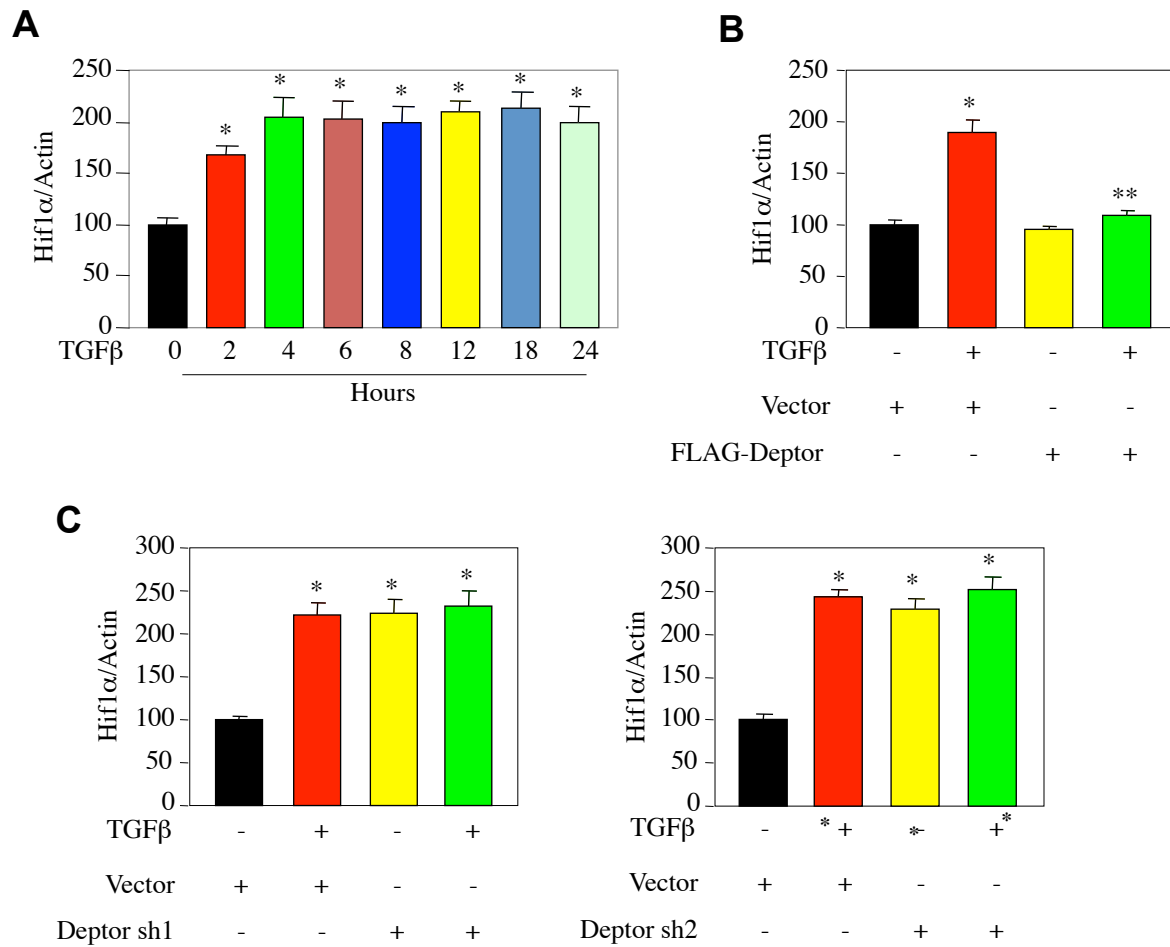

Supplement: Figure S7 — Quantification of the results shown in Fig. 4 . (A) Ratio of Hif1α to actin. Mean ± SE of 3 independent experiments is shown. For increase in 2 hours, *p<0.05 vs 0 hour; for increase in 4–24 hours *p<0.01 vs 0 hour. (B) Ratio of Hif1α to actin. Mean ± SE of 4 independent experiments is shown. *p<0.001 vs vector; **p<0.001 vs TGFβ-treated. (C) Ratio of Hif1α to actin. Mean ± SE of 4 independent experiments is shown. *p<0.05 vs vector alone for left panel; *p<0.001 vs vector for the right panel. (PDF) [file pone.0109608.s007.pdf]

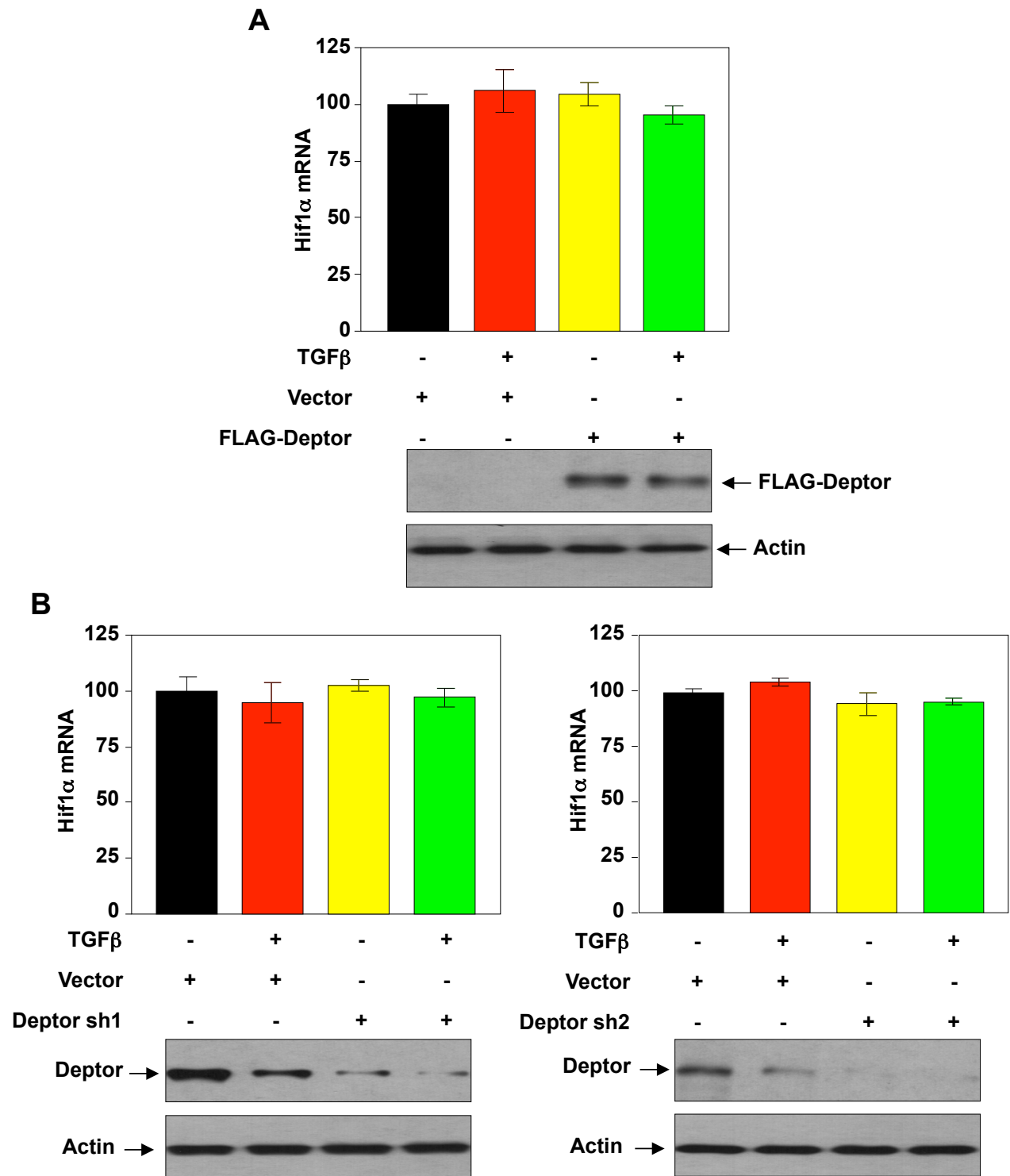

Supplement: Figure S8 — TGFβ does not regulate Hif1α mRNA expression. Human proximal tubular epithelial cells were transfected with FLAG-tagged Deptor expression vector (panel A) or Deptor sh1 or sh2 (panel B) as indicated followed by incubation with 2 ng/ml TGFβ for 24 hours. Expression of Hif1α mRNA was determined by real time RT-PCR as described in the Materials and Methods. Mean ± SE of triplicate measurements is shown. Bottom panels show FLAG-tagged deptor (panel A), deptor (panel B) and actin expression in parallel samples. (PDF) [file pone.0109608.s008.pdf]

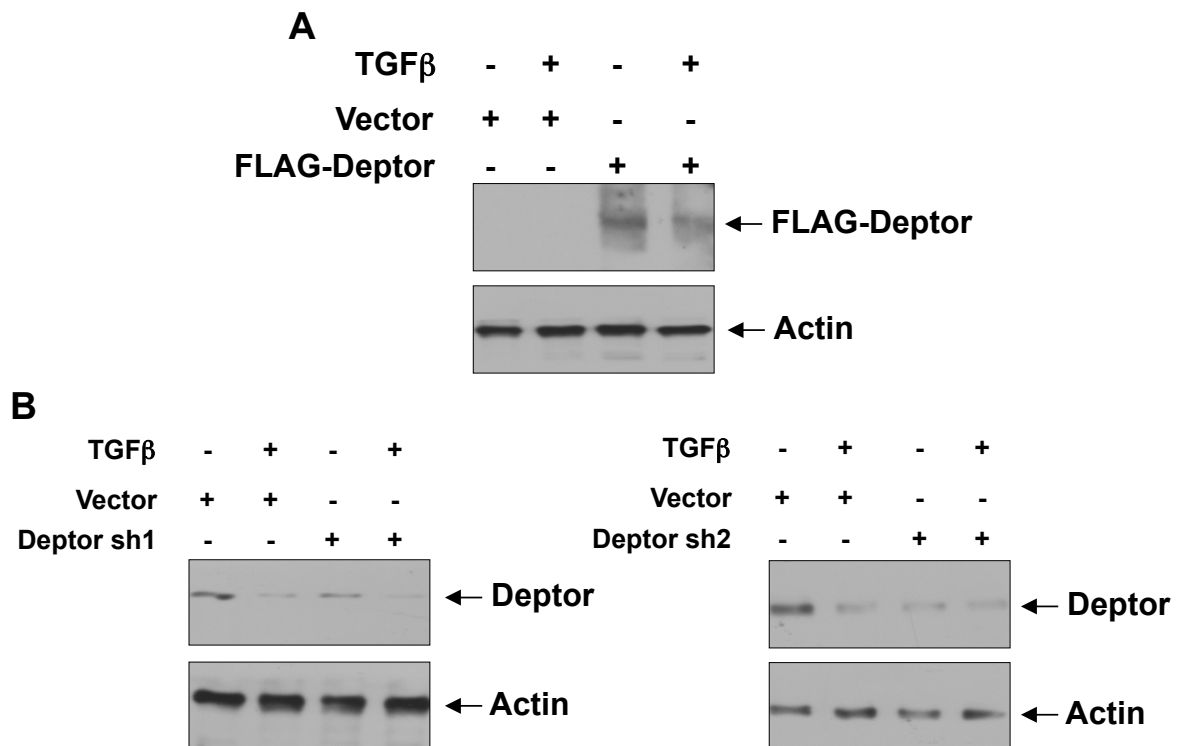

Supplement: Figure S9 — Expression of deptor for the results shown in Figure 5 . Human proximal tubular epithelial cells were transfected with expression vectors containing FLAG-Deptor (Panel A) or shRNAs against deptor (Panel B) prior to incubation with TGFβ as described in the legend of Fig. 5. The cell lysates were immunoblotted with FLAG and actin antibodies (Panel A) and deptor and actin antibodies (Panel B). (PDF) [file pone.0109608.s009.pdf]

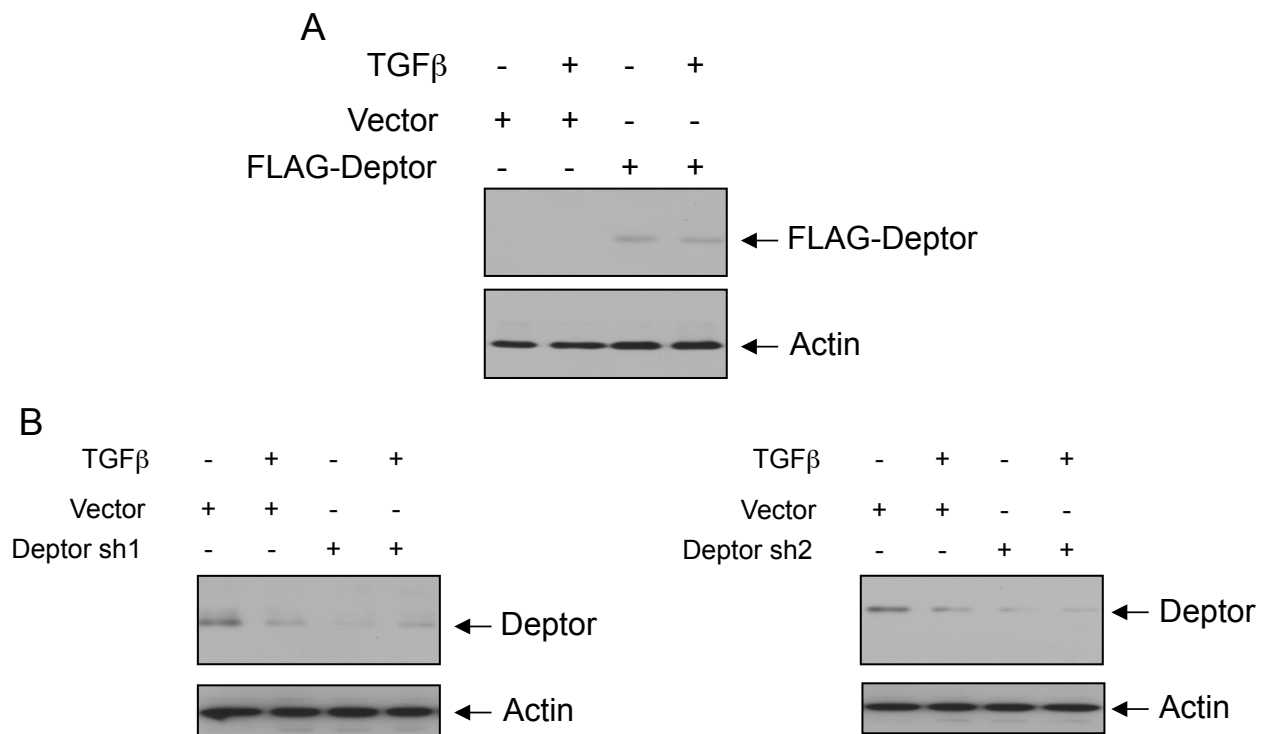

Supplement: Figure S10 — Expression of deptor for the results shown in Figure 6C and 6D . Human proximal tubular epithelial cells were transfected with expression vectors containing FLAG-Deptor (Panel A) as described in Fig. 6C or shRNAs against deptor (Panel B) as described in Fig. 6D prior to incubation with TGFβ. The cell lysates were immunoblotted with FLAG and actin antibodies (Panel A) and deptor and actin antibodies (Panel B). (PDF) [file pone.0109608.s010.pdf]

**A**

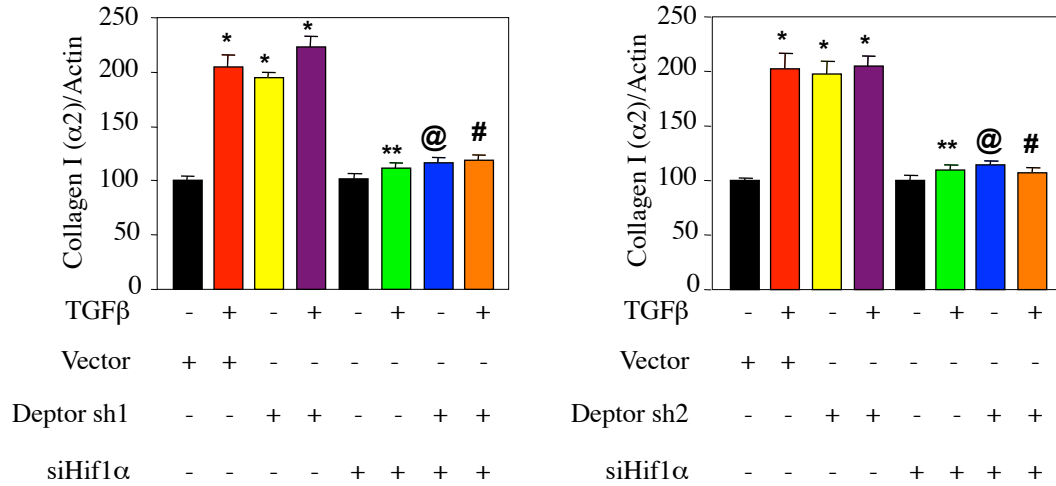

**B**

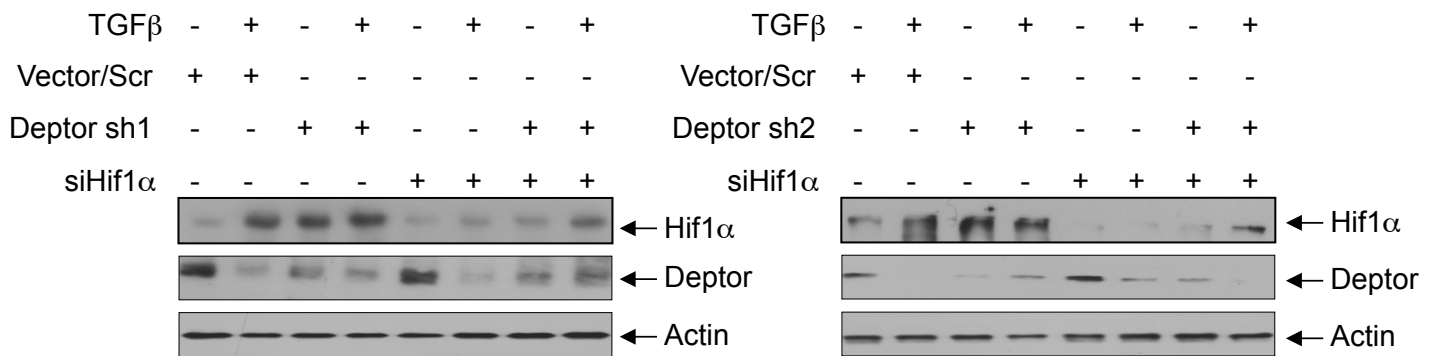

Supplement: Figure S11 — Quantification of the results shown in Fig. 7A . (A) Ratio of collagen I (α2) to actin. Mean ± SE of 4 independent experiments is shown. *p<0.001 vs vector alone. **p, @p, #p<0.001 vs TGFβ, shDeptor and shDeptor plus TGFβ, respectively. (B) Expression of deptor and Hif1α for the results shown in Figure 7B. Human proximal tubular epithelial cells were transfected with vector or scramble RNA (Scr) or shRNAs against deptor (Deptor sh1 and Deptor sh2) along with siRNA against Hif1α prior to incubation with TGFβ as described in the legend of Fig.7B. The cell lysates were immunoblotted with deptor, Hif1α and actin antibodies. (PDF) [file pone.0109608.s011.pdf]

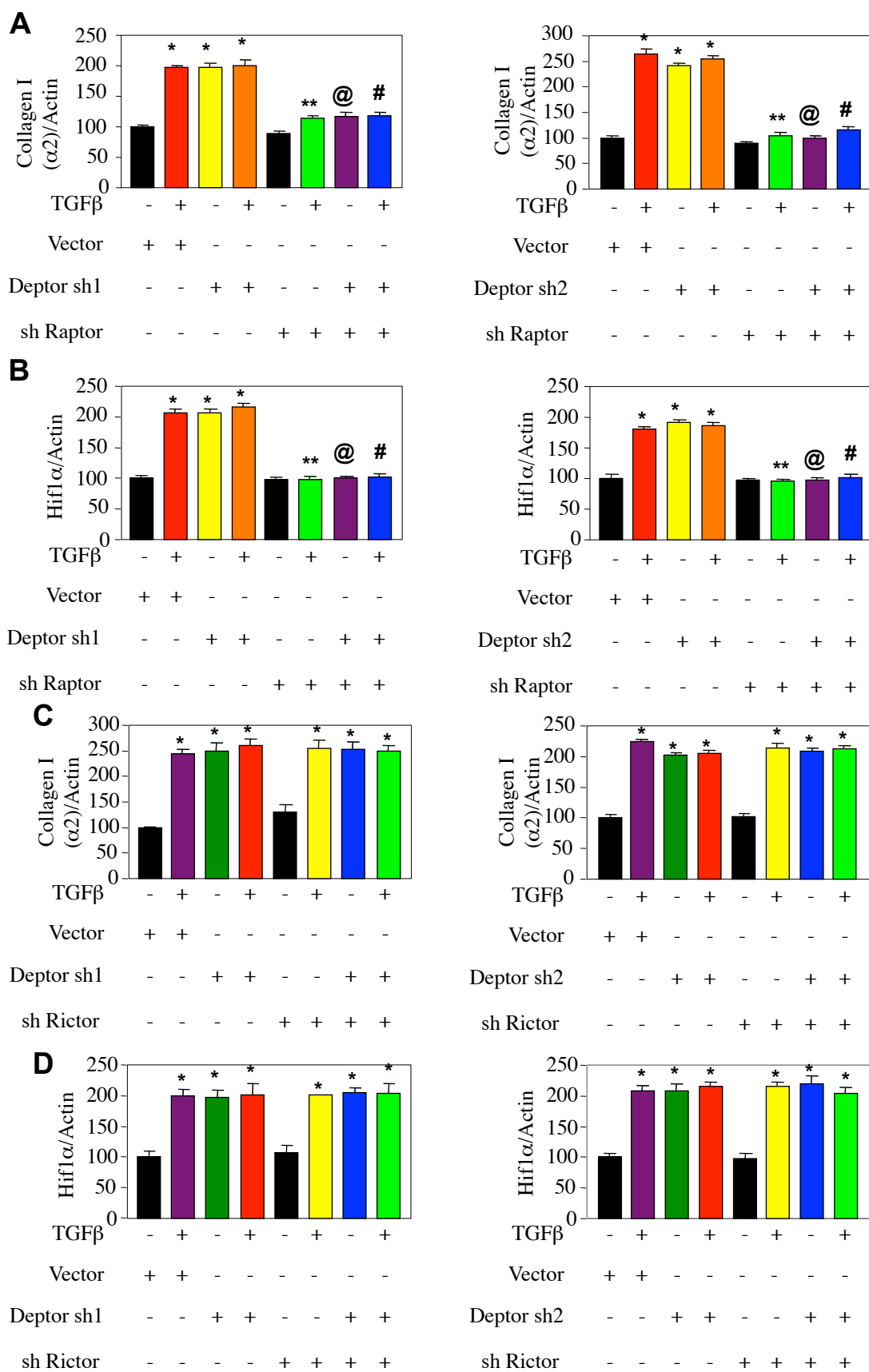

Supplementary Fig. S12  
Das F. et al

Supplement: Figure S12 — Quantification of the results shown in Fig. 8 . Ratios of collagen I (α2) to actin for Fig. 8A and 8C (panels A and C) and ratio of Hif1α to actin for Fig. 8B and 8D (panels B and D) are shown. Means ± SE of 4 independent experiments are shown for A–C and for left panel of D. For panel D right panel, mean ± SE of 5 experiments is shown. For panels A and B, *p<0.001 vs vector alone. **p, @p, #p<0.001 vs TGFβ, shDeptor and shDeptor plus TGFβ, respectively. For panels C and D, *p<0.001 vs vector alone. (PDF) [file pone.0109608.s012.pdf]

**A**

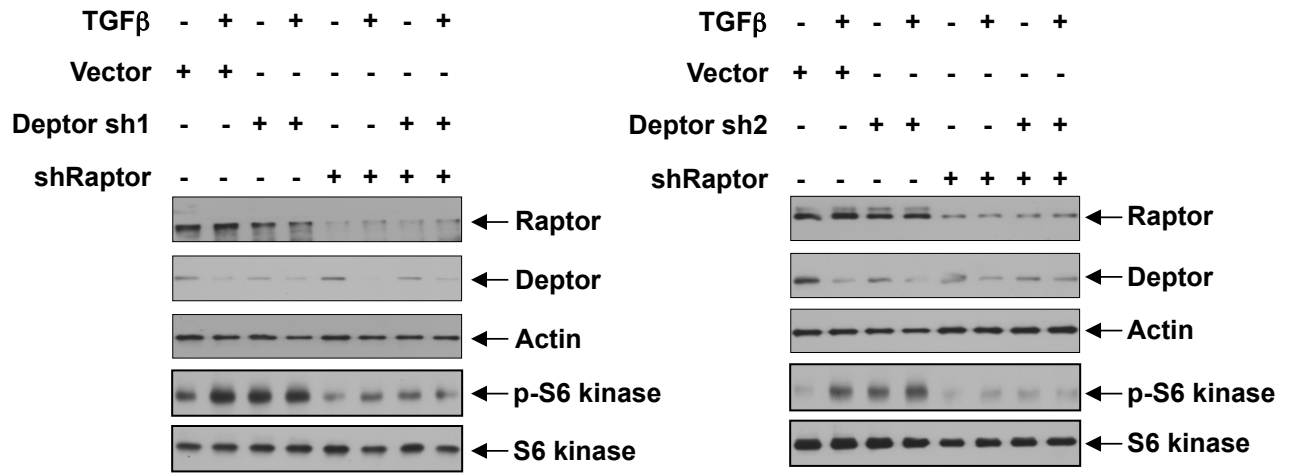

**B**

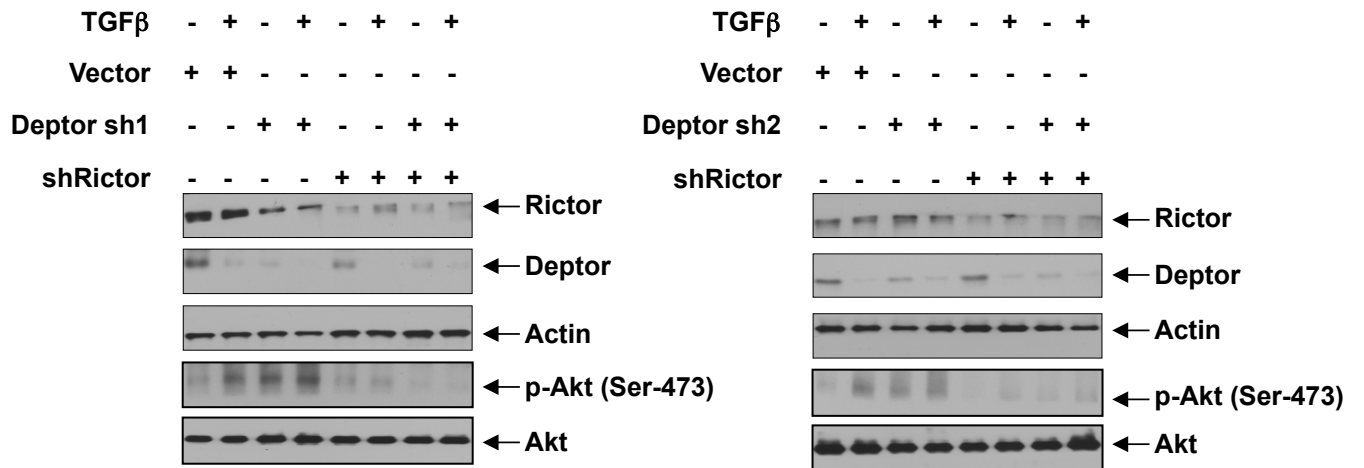

Supplement: Figure S13 — Expression of raptor, rictor, deptor, and activation of mTORC1 (phospho-S6 kinase) and activation of mTORC2 (phosphorylation of Akt at Ser-473) for the results shown in Figure 9 . Human proximal tubular epithelial cells were transfected with vector or deptor shRNA expression plasmids along with raptor shRNA (Panel A) or rictor shRNA (Panel B) prior to incubation with TGFβ as described in the legend of Fig. 9. The cell lysates were immunoblotted against raptor, phospho-S6 kinase (Thr-389), S6 kinase (panel A), rictor, phospho-Akt (Ser-473), Akt (Panel B), deptor and actin antibodies as indicated. (PDF) [file pone.0109608.s013.pdf]

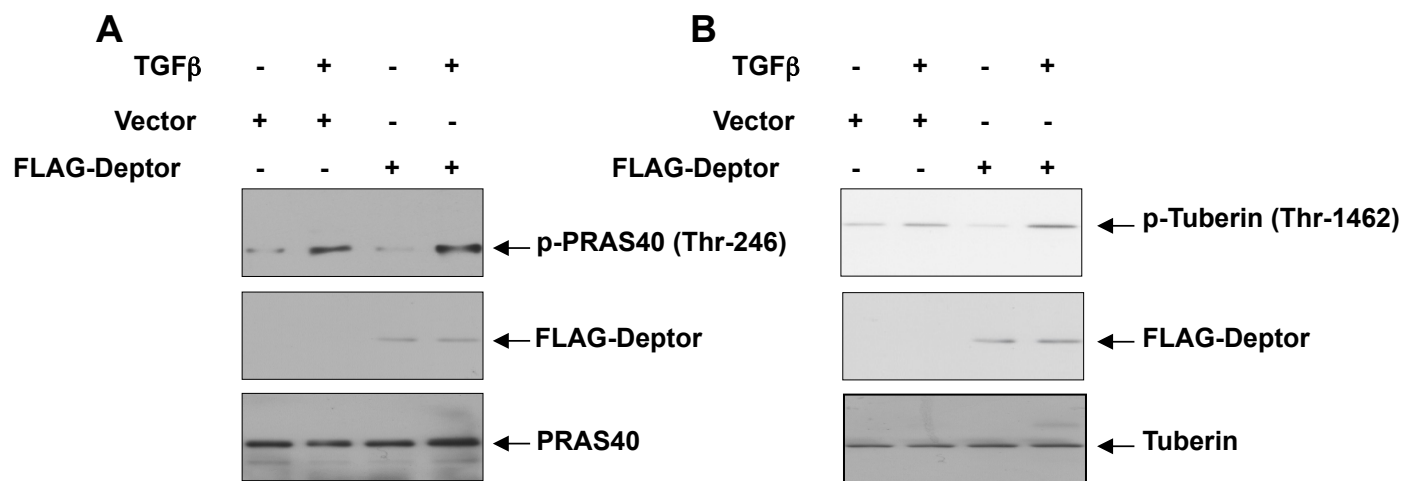

Supplement: Figure S14 — Expression of deptor does not inhibit rapid phosphorylation of Akt substrates PRAS40 and tuberin in response to TGFβ. Human proximal tubular epithelial cells were transfected with vector or FLAG-Deptor. The transfected cells were incubated with 2 ng/ml TGFβ for 15 minutes. The cell lysates were immunoblotted with phospho-PRAS40 (Thr-246), PRAS40 (Panel A) and phospho-tuberin (Thr-1462), tuberin (Panel B) antibodies. Expression of deptor was detected by FLAG immunoblot. (PDF) [file pone.0109608.s014.pdf]

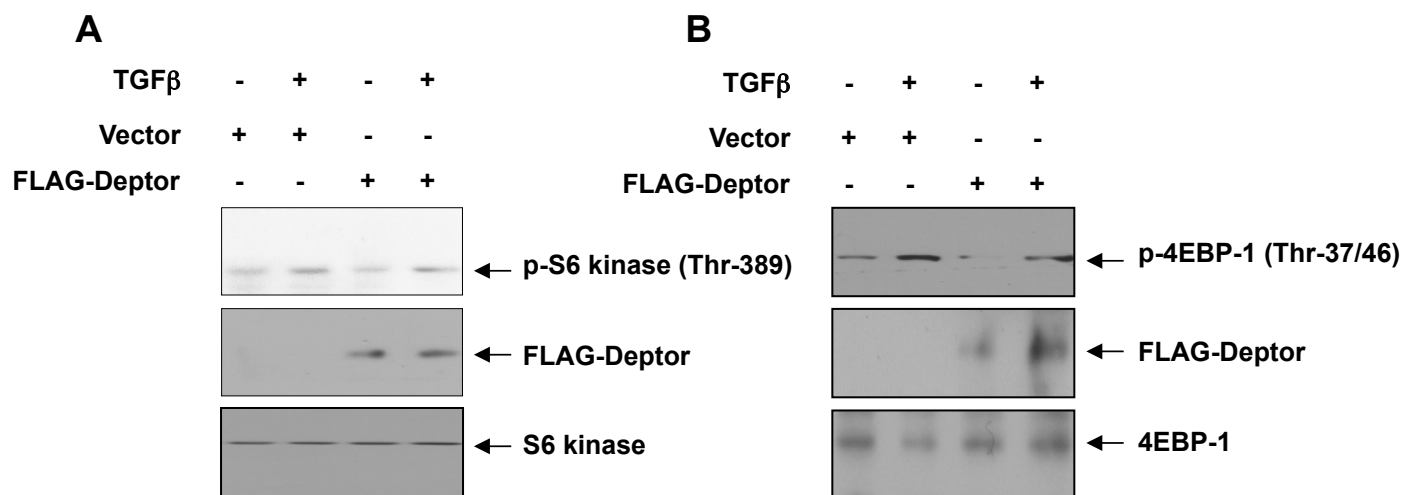

Supplement: Figure S15 — Expression of deptor does not inhibit rapid activation of mTORC1 in response to TGFβ. Human proximal tubular epithelial cells were transfected with vector or FLAG-Deptor. The transfected cells were incubated with 2 ng/ml TGFβ for 15 minutes. The cell lysates were immunoblotted with antibodies for phospho-S6 kinase (Thr-389) (panel A) and phospho-4EBP-1 (Thr-37/46) (panel B) as indicators of mTORC1 activation. The lysates were also immunoblotted with FLAG antibody and S6 kinase (Panel A) and 4EBP-1 (Panel B) antibodies. (PDF) [file pone.0109608.s015.pdf]

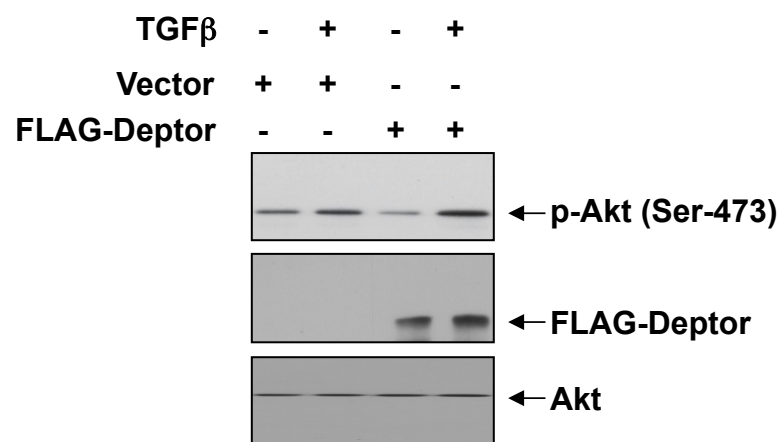

Supplement: Figure S16 — Expression of deptor does not inhibit rapid activation of mTORC2. Human proximal tubular epithelial cells were transfected with vector or FLAG-Deptor. The transfected cells were incubated with 2 ng/ml TGFβ for 15 minutes. The cell lysates were immunoblotted with phospho-Akt (Ser-473) antibody as indicator of mTORC2 activation. The lysates were also immunoblotted with FLAG and Akt antibodies. (PDF) [file pone.0109608.s016.pdf]

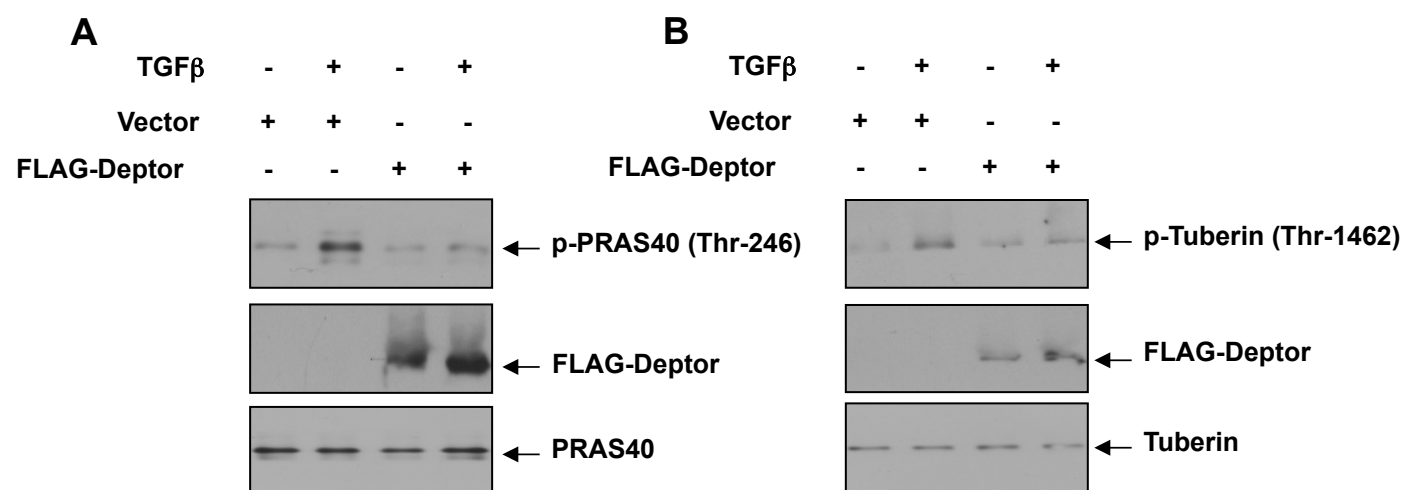

Supplement: Figure S17 — Expression of deptor inhibits phosphorylation of Akt substrates PRAS40 and tuberin in response to prolonged TGFβ incubation. Human proximal tubular epithelial cells were transfected with vector or FLAG-Deptor. The transfected cells were incubated with 2 ng/ml TGFβ for 24 hours. The cell lysates were immunoblotted with phospho-PRAS40 (Thr-246), PRAS40 (Panel A) and phospho-tuberin (Thr-1462), tuberin (Panel B) antibodies. Expression of deptor was detected by FLAG immunoblot. (PDF) [file pone.0109608.s017.pdf]
