# Supplementary material for: TGFβ-Induced Deptor Suppression Recruits mTORC1 and Not mTORC2 to Enhance Collagen I (α2) Gene Expression
Source: PLoS One. 2014 Oct 15;9(10):e109608. doi: 10.1371/journal.pone.0109608 (PMC4198127; doi:10.1371/journal.pone.0109608)
Supplement: Table S1 — List of antibodies used in this study. (PDF) [file pone.0109608.s018.pdf]

Supplementary Table 1

Antigen	Species	Vendor	Cat. No
Deptor	goat polyclonal	Santa Cruz Biotechnology	Sc-87692
Hif 1α	rabbit polyclonal		Sc-10790
Collagen 1 (α2)	mouse monoclonal		Sc-166865
Tuberin	rabbit polyclonal		Sc-893
phospho-S6 kinase (Thr-389)	rabbit polyclonal	Cell Signaling Technologies	9205
S6 kinase	rabbit polyclonal		9202
phosphor-4EBP1 (Thr-37/46)	rabbit polyclonal		9459
4EBP-1	rabbit polyclonal		9452
Phosphor-Akt (Ser-473)	rabbit polyclonal		9271
Phosphor-Akt (Thr-308)	rabbit polyclonal		9275
Akt	rabbit polyclonal		9272
Raptor	rabbit monoclonal		2280
Rictor	rabbit polyclonal		2140
phosphor- PRAS40 (Thr-246)	rabbit monoclonal		2997
PRAS40	rabbit monoclonal		2691
Phosphor Tuberin/TSC2 (Thr -1462)	rabbit polyclonal		3611
Actin	rabbit polyclonal	Sigma	A-2066
Flag	mouse monoclonal		F-4042
